# BASP1 labels neural stem cells in the neurogenic niches of mammalian brain

**DOI:** 10.1038/s41598-021-85129-1

**Published:** 2021-03-10

**Authors:** Louis N. Manganas, Irene Durá, Sivan Osenberg, Fatih Semerci, Mehmet Tosun, Rachana Mishra, Luke Parkitny, Juan M. Encinas, Mirjana Maletic-Savatic

**Affiliations:** 1 Department of Neurology, Stony Brook University Medical Center, Stony Brook, NY USA; 2 Achucarro Basque Center for Neuroscience, Leioa, Spain; 3 The Basque Foundation for Science, IKERBASQUE, Bilbao, Spain; 4 Department of Neuroscience, University of the Basque Country (UPV/EHU), Leioa, Spain; 5 Departments of Pediatrics, Neurology and Neuroscience, Baylor College of Medicine, Houston, TX USA; 6 Jan and Dan Duncan Neurological Research Institute at Texas Children’s Hospital, Houston, TX USA; 7 Department of Neurology, Stony Brook University Medical Center, Health Sciences Center T-12, room 020, Stony Brook, NY 11794 USA; 8 Departments of Pediatrics, Neurology, and Neuroscience, Baylor College of Medicine, Jan and Dan Duncan Neurological Research Institute at Texas Children Hospital, 1250 Moursund St., Rm 1250, Houston, TX 77030 USA

**Keywords:** Neuroscience, Stem cells

## Abstract

The mechanisms responsible for determining neural stem cell fate are numerous and complex. To begin to identify the specific components involved in these processes, we generated several mouse neural stem cell (NSC) antibodies against cultured mouse embryonic neurospheres. Our immunohistochemical data showed that the NSC-6 antibody recognized NSCs in the developing and postnatal murine brains as well as in human brain organoids. Mass spectrometry revealed the identity of the NSC-6 epitope as brain abundant, membrane-attached signal protein 1 (BASP1), a signaling protein that plays a key role in neurite outgrowth and plasticity. Western blot analysis using the NSC-6 antibody demonstrated multiple BASP1 isoforms with varying degrees of expression and correlating with distinct developmental stages. Herein, we describe the expression of BASP1 in NSCs in the developing and postnatal mammalian brains and human brain organoids, and demonstrate that the NSC-6 antibody may be a useful marker of these cells.

## Introduction

To determine the potential of neural stem and progenitor cells as therapeutic agents, the intricate pathways that mediate their proliferation, survival, and differentiation must be understood^1–3^. To accomplish this goal, we need selective markers that would allow specific studies of the heterogeneous population of neural stem and progenitor cells (collectively labeled NPCs). Existing markers such as nestin, Sox-2, brain lipid-binding protein (BLBP), glial fibrillary acidic protein (GFAP) and others, are expressed not only by NPCs but also by other cell types, mainly of the astroglial lineage^4–9^. While several other markers appear to be selective for NSCs^10–13^, they are all intracellular, limiting characterization of these cells to ex-vivo studies. To identify novel markers of these cells and capture possible membrane-bound antigens, we generated several mouse antibodies (Abs) against cultured mouse embryonic neurospheres that contain both neural stem and progenitor cells. The NSC-6 Ab, which produced the most robust staining of NPCs, corresponded to Brain-Abundant, membrane-attached Signal Protein 1 (BASP1), a protein not previously described in NPCs.

BASP1 (also known as *NAP-22* and *CAP-23*) belongs to a family of growth-associated proteins, which include growth-associated protein 43 (GAP-43) and myristoylated alanine-rich protein kinase C substrate (MARCKS). It is considered a signal processing protein that plays critical roles in synaptic plasticity and neurite outgrowth^14–19^. It is a hydrophilic 23 kDa protein with SDS-PAGE electrophoresis mobility of 58 kDa^20^. BASP1 also contains a basic domain that allows binding of phosphatidylinositol-4, 5-bisphosphate (PIP_2_) and calmodulin, regulated by protein kinase C (PKC)-mediated phosphorylation on Ser-5^21^. In turn, its hydrophobic properties through N-myristoylation are critical for plasma membrane^20^, PKC, and calmodulin binding^21^. During development, BASP1 accounts for almost 1% of the total protein in brain and almost half of that in the adult brain^22,23^ thus supporting a role for synapse formation during development and synaptic function in adulthood. Not surprisingly, BASP1 mouse gene knockouts are non-viable^18^. In contrast, BASP1 over-expression in adult neurons^24^ and in PC12E2 cells and hippocampal neurons^25^ stimulates neurite outgrowth. This effect is believed to occur independently of the neural cell adhesion molecule (NCAM) pathway^26^ and to rely on precise plasma membrane localization and organization^25^. In the adult brain, BASP1 is expressed in the cerebral cortex, hippocampus, olfactory bulb, basal ganglia, thalamus and cerebellum^27^. In addition to plasma membrane, it has been reported, similarly to GAP-43, MARCKS and CAP-23, in the cytoplasm^28–30^ and nucleus^31,32^. It is mainly distributed to the synaptic terminals, dendritic spines, and synaptic vesicles, suggesting an important role in synaptic function^33^.

BASP1 has not been reported in NPCs and thus, our finding that an NPC-derived antibody labels this protein led us to pursue detailed characterization of BASP1 in the developing and adult mammalian neurogenic regions. During development, BASP1 is expressed throughout the brain, while in adulthood, it is restricted to neurogenic regions. Interestingly, in the adult hippocampal niche, it is restricted to type I, radial neural stem cells (NSCs) while in the subventricular niche, it is limited to B and C cells and GFAP-expressing cells in the rostral migratory stream (RMS)—these likely represent astrocytic tubes through which C cells migrate. Overall, our results suggest that BASP1 has potential value as a marker of NSCs.

## Results

### NSC-6 stains mouse and human NPCs

**Figure 1:**
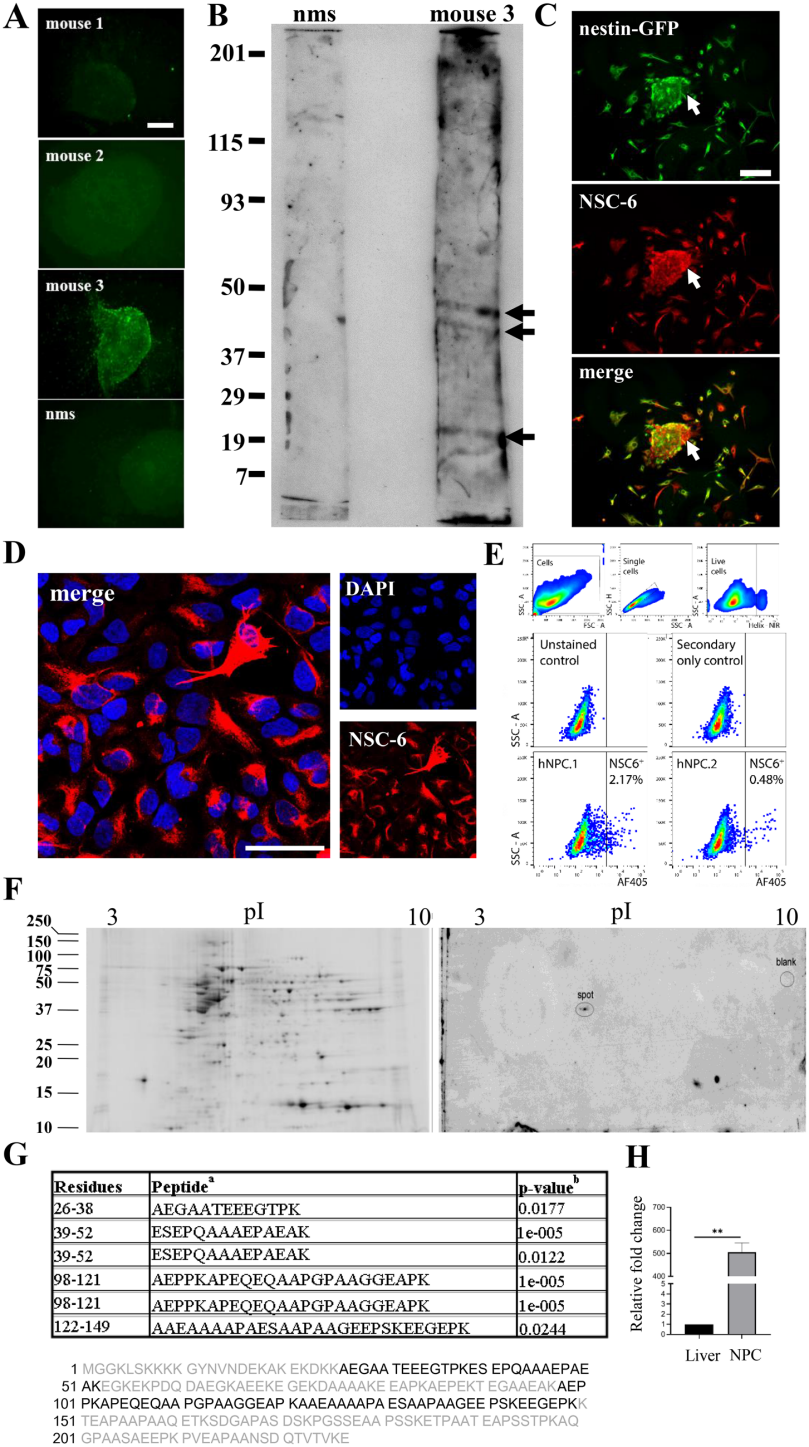
Mouse-derived polyclonal antibody against mouse neurospheres identifies BASP1 as its antigen. (**A**) Neurospheres immunolabeled with tail bleeds from mice 1, 2, and 3, and normal mouse serum (nms) (all at 1:1000). (**B**) Immunoblot of the neurosphere lysate with mouse 3 tail bleed and nms (both at 1:10). The arrows indicate three distinct bands of 49 kDa, 46 kDa and 22 kDa. Full length strips are shown in a single cropped image. (**C**) Epifluorescent images of *Nestin*-GFP neurospheres (green) labeled with NSC-6 antibody (red) show high immunolabeling of the neurosphere (merged, yellow). All the *Nestin*-GFP cells are NSC-6 positive, but some NSC-6 immunopositive cells are negative for *Nestin*-GFP (arrow). Scale bar is 100 µm in A and 50 µm in C. (**D**) Human neural progenitor cells (hNPCs) labelled with DAPI (blue) and NSC-6 antibody (1:20, red). Scale bar is 20 µm. (**E**) Sorting of hNPCs stained with unconjugated NSC-6 antibody and Day Light 405 as the secondary antibody. 250,000 live cell events were acquired. Single viable cell gating was carried out as shown in upper panels. Exclusion of NSC-6^neg^ and false-positive signals was done by gating outside unstained cells and cells stained with the secondary antibody only (middle panels). Two independently generated hNPC lines (hNPC.1 and hNPC.2) were examined, showing a subpopulation of NSC-6 labeled cells. (**F**) *Left* A 2-DE gel obtained with 90 µg of human hippocampus lysate and stained for total proteins with Sypro Ruby fluorescent stain. *Right* Immunoblot obtained from the same protein load run on an identical gel in parallel with that of (**B**), and immunolabeled with the NSC-6 Ab. The spot of interest, corresponding to molecular weight of 45 kDa, is indicated in the center of the blot. The location of a blank, control spot is also indicated. Separate blots, shown in their entirety, were cropped and separated by white space. (**G**) LC/MS/MS analysis indicates that the spot of interest is accession # IPI00299024 BASP1, brain abundant, membrane attached signal protein 1. Four peptides were identified in six spectra, with 34.9% coverage. ^a^Each peptide sequence was determined in distinct MS/MS spectra. All six spectra were manually confirmed. ^b^p-values < 0.025 were considered statistically acceptable. (**H**) RT-PCR of BASP1 mRNA in cultured adult mouse NPCs and mouse liver cells (negative control).

In this study, we generated several mouse Abs against mouse NPCs and characterized one Ab in particular, NSC-6. Initial screening of tail bleeds obtained from each of the three mice immunized with NPCs revealed that one mouse (number 3) produced the most robust immunolabeling against cultured neurospheres, while tail bleeds from mice 1 and 2 showed little or no immunolabeling, comparable to that obtained with wild-type non-immunized mouse serum (Fig. 1A). Immunoblot analysis using the tail bleed from mouse number 3 against protein extracts isolated from NPCs revealed multiple bands at 49 kDa, 46 kDa and 22 kDa (Fig. 1B), while immunoblots using tail bleeds from mice 1 or 2 did not display any specific immunoreactivity and were similar to non-immunized mouse serum. Based on these consistent immunoblot and immunocytochemical results, we used mouse number 3 as a source for splenocytes to generate hybridomas. As described in the “Materials and methods” section, one Ab, NSC-6, yielded robust signal by ELISAs performed against neurospheres and was selected for further analyses. Of note, we could not isolate a monoclonal clone despite several rounds of NSC-6 hybridoma subcloning. It is unclear whether further subcloning is necessary or fusion with more than one lymphocyte occurred during hybridoma production.

To validate the specificity of NSC-6 immunolabeling against NPCs, we cultured neurospheres from *Nestin*-GFP transgenic mice in which the green fluorescent protein (GFP) expression is driven by the nestin regulatory elements^5^. Nestin is a specific marker of neuroepithelial stem cells^34^. Cultured neurospheres, generated from *Nestin*-GFP embryos, were immunolabeled with NSC-6 (Fig. 1C). All *Nestin*-GFP expressing cells were positive for NSC-6 immunolabeling. NSC-6 also immunolabeled a minority of cells adhering to the plate that exhibited low or undetectable levels of *Nestin*-GFP. In addition, we derived human neuroprogenitor cells (hNPCs) from induced pluripotent stem cells (iPSCs) obtained from a healthy adult and stained them with NSC-6. The NSC-6 labeled a portion of hNPCs (Fig. 1D), suggesting different populations of hNPCs in our culture. Indeed, sorting of the hNPCs labeled with the NSC-6 confirmed the existence of NSC-6 positive and negative population in two independent experiments (Fig. 1E).

### NSC-6 antibody recognizes BASP1

To determine the identity of the NSC-6 antigen, we employed Liquid Chromatography–Mass Spectrometry (LC–MS) with peptide mass fingerprinting or LC–MS/MS (tandem MS) on the spot excised from the 2D gel containing human hippocampal extract (Fig. 1F). We observed four distinct, statistically acceptable peptide sequences in six MS/MS spectra, accounting for 34.8% coverage of the protein BASP1 (Fig. 1G). As expected, the excised spot contained an abundance of bovine proteins (casein and albumin) resulting from the immunoblotting protocol. The only detectable human proteins were a small variety of keratins (common contaminants observed in LC/MS/MS studies) and the brain abundant, membrane attached signal protein 1 (BASP1). As control, we excised a similarly sized piece of blot from the margin not exposed to the gel-protein transfer but treated to the immunostaining process. We observed all bovine proteins and human keratins in the control spot digest, but there was no evidence of BASP1 in this sample (Fig. 1F, right panel). To then confirm the BASP1 expression in adult mouse neurospheres, we performed RT-PCR and observed significant difference between BASP-1 mRNA in mouse NPCs compared to mouse liver, used as a negative control (Fig. 1H).

### The NSC-6 antibody localizes BASP1 to radial neural stem cells (NSCs) in the embryonic mouse brain

**Figure 2:**
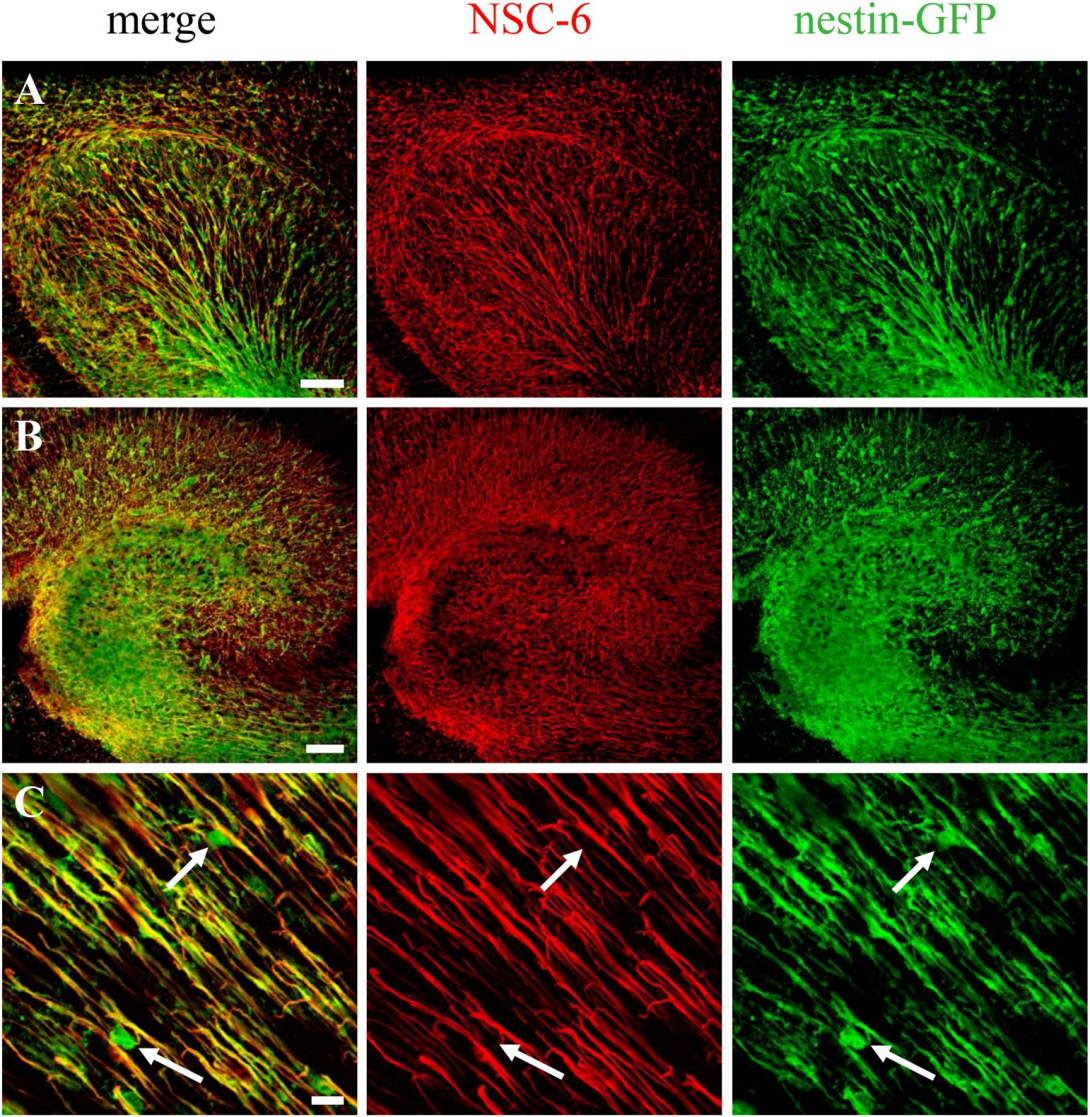
NSC-6 antibody immunolabels radial glia in the embryonic mouse brain. NSC-6 immunolabeling (red) is present along the processes of radial glia in *Nestin*-GFP embryonic (E12) cortex (**A**) and hippocampus (**B**). (**C**) High magnification image of the embryonic cortex shows *Nestin*-GFP expression in the nucleus and soma of radial glia (arrows). These compartments were not immunolabeled by the NSC-6 Ab. Scale bars are 50 µm in (**A**,**B**); and 5 µm in (**C**).

To determine whether BASP1 is expressed in NSCs of the developing mouse brain, we immunolabeled brain sections from embryonic day 12 (E12) *Nestin*-GFP mice with the NSC-6 Ab (Fig. 2). During embryonic development of the cerebral cortex, radial glia not only serve as scaffolds for migrating neuroblasts, but also as precursor cells that generate neurons and glia^35^. Radial glia expressing *Nestin*-GFP were localized throughout the developing embryonic brain (Fig. 2), including cortex (Fig. 2A) and the future hippocampal region (Fig. 2B). All cells expressing *Nestin*-GFP also exhibited NSC-6 immunolabeling, present on the GFP-positive radial glia longitudinal processes (Fig. 2C). Furthermore, Sox2 staining confirmed that NSC-6 did not identify amplifying neuroprogenitors in the subventricular zone at E12 (Supplementary Fig. S1). These results suggest that BASP1, recognized by NSC-6 Ab, is expressed in radial glia during embryonic brain development.

### The NSC-6 antibody localizes BASP1 to known neurogenic regions in the postnatal mouse brain

**Figure 3:**
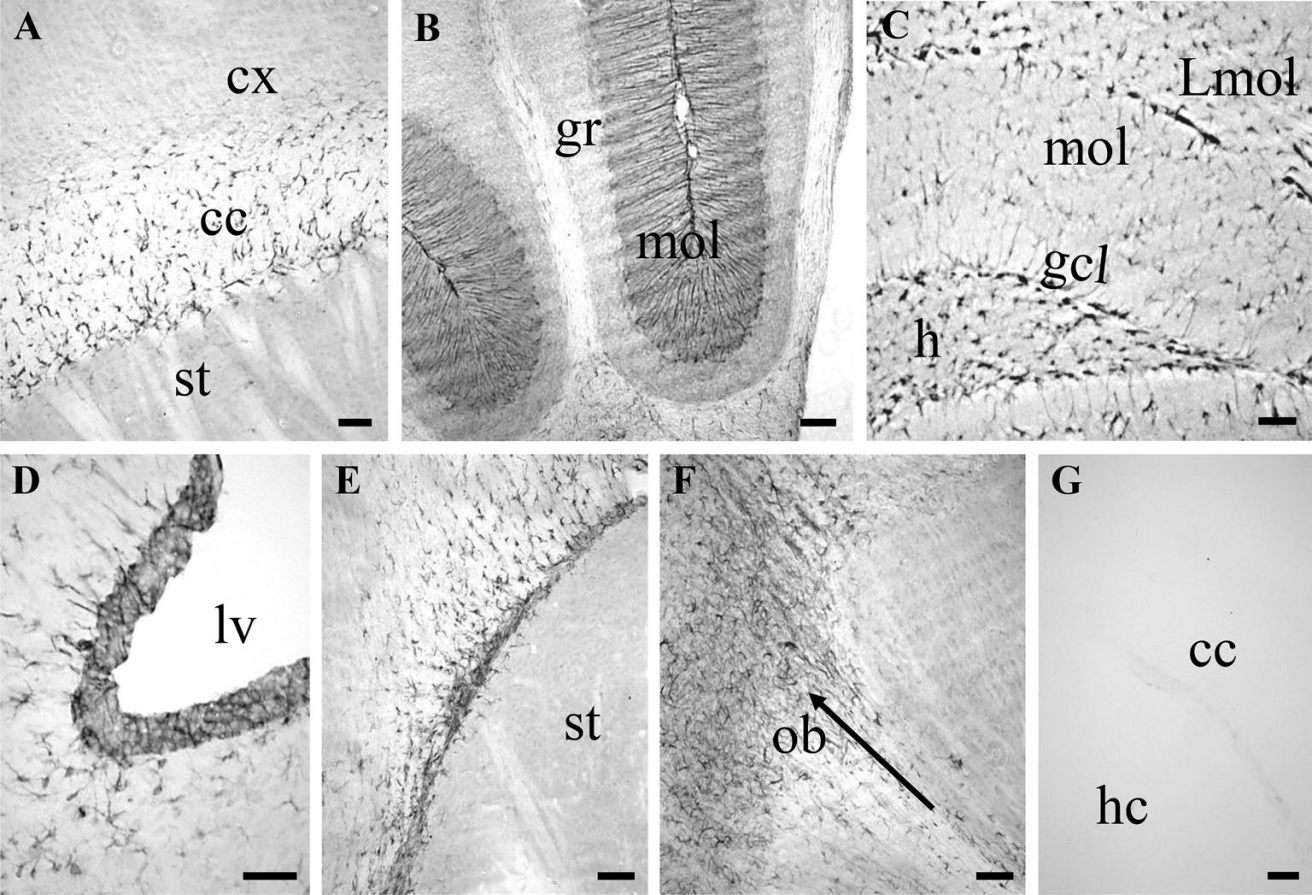
NSC-6 antibody immunolabels different adult mouse brain regions. (**A**) NSC-6 immunopositive glia-like cells in the corpus callosum (cc). Note the absence of NSC-6 immunolabeling in the cortex (cx) and the striatum (st). (**B**) NSC-6 immunolabeling of Bergmann glia processes in the molecular layer (mol) of the cerebellum. The granular layer (gr) lacks NSC-6 immunolabeling. (**C**) NSC-6 labels sparse astrocytes-like cells in the hilus (h), the molecular layer (mol), and the lacunosum moleculare layer (Lmol) of the dentate gyrus. Radial-astrocyte processes—most likely belonging to NSCs due to their perpendicular orientation—in the granule cell layer (gcl) are also immunolabeled. (**D**) NSC-6 immunolabeling of the SVZ adjacent to the lateral ventricle (lv) and surrounding parenchyma. (**E**) NSC-6 immunolabeling of the RMS. Note the absence of staining in the striatum (st). (**F**) NSC-6 immunolabeling of the rostral aspect of RMS, entering and disseminating in the olfactory bulb (ob). The arrow points the rostral direction. (**G**) Omission of the primary antibody resulted in absence of detectable immunolabeling throughout the entire brain. The corpus callosum between the cortex (cx) and hippocampus (hc) is shown. Scale bars are 50 µm in all images.

To characterize BASP1 expression in the postnatal mouse brain, we carried out diaminobenzidine (DAB)-based immunolabeling in 4-week old mice (Fig. 3). In general, NSC-6 Ab immunolabeled neurogenic areas of the postnatal mouse brain—presumably NPCs—and cells in the white matter such as the corpus callosum (Fig. 3A), the anterior commissure, and the cerebellum, where NSC-6 immunolabeling was present in the Bergmann glia radial processes in the molecular layer (Fig. 3B). NSC-6-immunopositive cells were also found in the hilus, the granule cell layer, and the molecular layer of the dentate gyrus (Fig. 3C). Remarkably, robust staining was found in presumptive NSCs of the subgranular zone (SGZ), known to harbor the neurogenic stem cells of the adult hippocampus^36^. In addition, NSC-6 immunolabeling was observed in the subventricular zone (SVZ) of the lateral ventricle (Fig. 3D), while both caudal (Fig. 3E) and rostral (Fig. 3F) parts of the RMS were conspicuously immunolabeled. NSC-6 immunolabeling was not observed in other brain regions, such as cortex and striatum (Fig. 3A,B,E). Omission of the primary Ab produced no immunolabeling (Fig. 3G).

### The NSC-6 antibody localizes BASP1 to NSCs in neurogenic niches of the postnatal mouse brain

**Figure 4:**
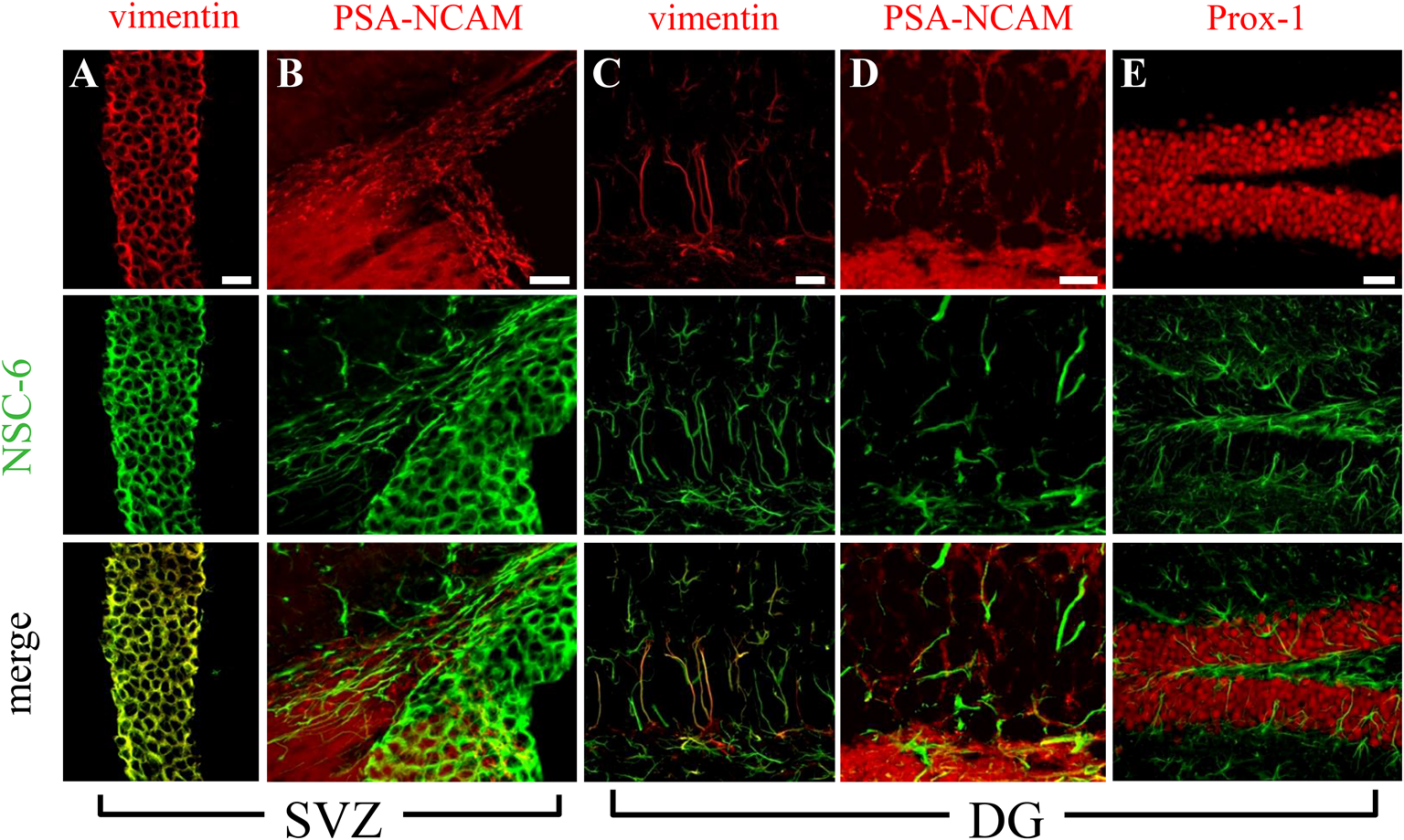
The NSC-6 antibody does not label neuronal lineages in the adult mouse neurogenic regions. NSC-6 immunolabeling colocalizes with vimentin (**A**) but not PSA-NCAM in the SVZ. (**B**) NSC-6 immunolabeling colocalizes with vimentin (**C**) but not PSA-NCAM (**D**) or Prox-1 (**E**) in the dentate gyrus (DG). Scale bars are 20 µm in (**A**–**C**); 10 µm in (**D**); and 40 µm in (**E**).

To further investigate the expression of BASP1, we double-immunolabeled brain sections with NSC-6 Ab and a panel of diagnostic cell markers (Fig. 4). In the SVZ and the dentate gyrus, NSC-6 immunolabeling colocalized with markers of NSCs, such as vimentin (Fig. 4A,C) and GFAP (discussed below). In contrast, it did not colocalize with markers of neuroblasts, immature, and mature neurons, such as PSA-NCAM (Fig. 4B,D), Prox-1 (Fig. 4E), and NeuN (Supplementary Fig. S2), respectively, as well as microglia (Iba-1), oligodendrocyte progenitors (NG2), or mature oligodendrocytes (myelin basic protein (MBP). These data confirm that BASP1expression, as defined by NSC-6 Ab immunolabeling, is restricted to the NSCs in both postnatal neurogenic niches.

### The NSC-6 antibody localizes BASP1 to NSCs and not ANPs in the mouse hippocampus

**Figure 5:**
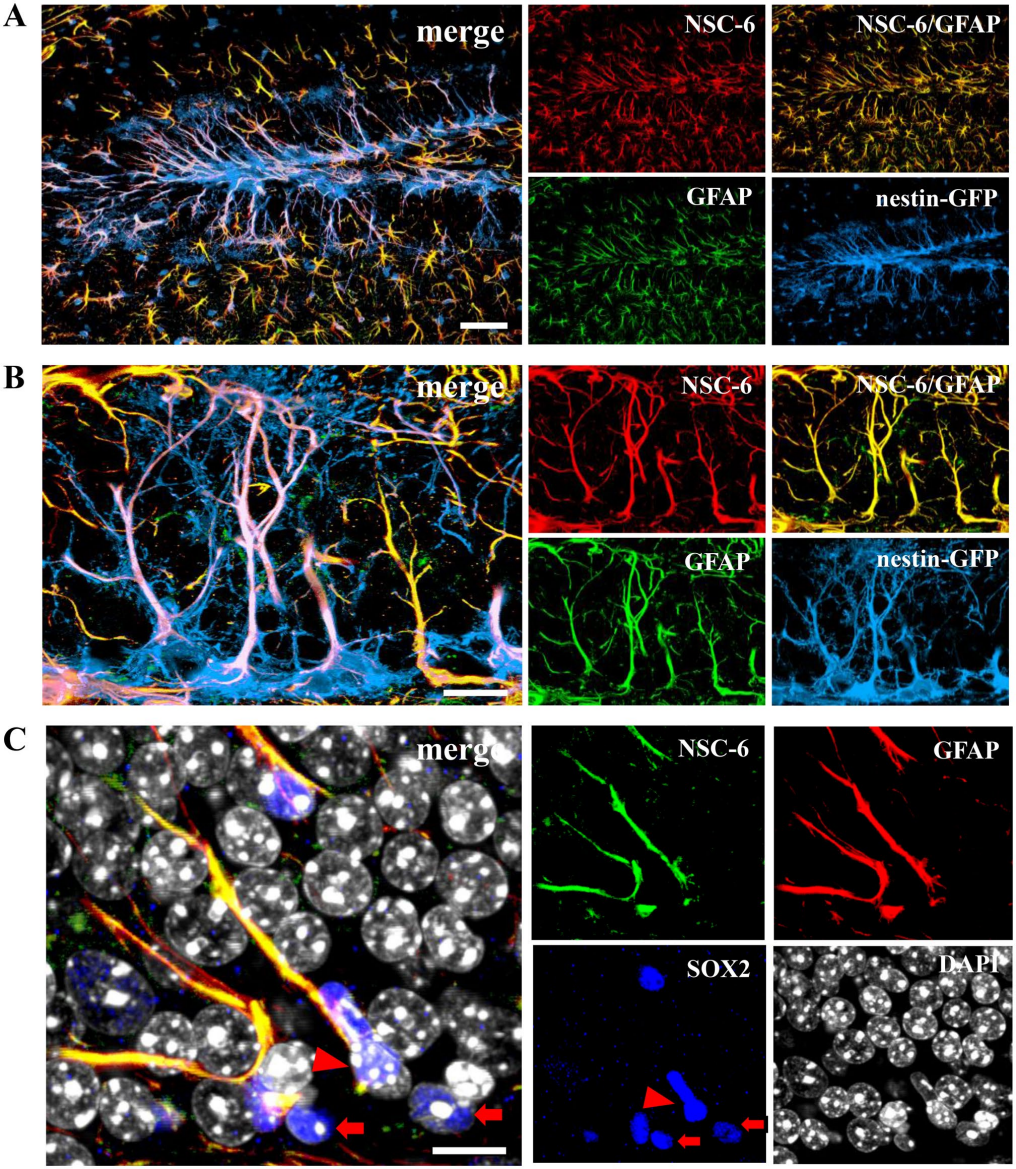
NSC-6 antibody immunolabels adult hippocampal NSCs but not ANPs. (**A**) NSC-6 immunolabeling co-localizes with GFAP in the hilus, the SGZ, the granular cell layer, and the molecular layer of the dentate gyrus. (**B**) NSC-6 immunolabeling co-localizes with GFAP and *Nestin*-GFP in the radial process of the NSCs located in the SGZ. (**C**) NSC-6 does not label Sox2+ GFAP− ANPs (arrows) adjacent to NSC-6+; GFAP+; Sox2+ NSCs (arrowhead). Scale bars are 25 µm in A, 5 µm in B, and 10 µm in (**C**).

In the adult hippocampus, neurogenesis begins with primary radial NSCs (type I cells), which express nestin, vimentin, GFAP, and BLBP in their apical processes^35,37–39^. These NSCs divide asymmetrically, giving rise to amplifying neuroprogenitors (ANPs, type II cells), which proliferate symmetrically before exiting cell cycle and differentiating slowly into neurons^35,36,39,40^. ANPs and NSCs differ in morphology and expression markers: ANPs are small, round cells that express low levels of nestin and lack vimentin and GFAP. In *Nestin*-GFP transgenic mice, NSC-6 Ab immunolabeling colocalized with GFAP, indicating that type I NSCs, and not type II ANPs, express BASP1 (Fig. 5A,B). In addition, we observed random and sparse NSC-6 and GFAP colocalization in the hilus and the hippocampal molecular layer, indicating that BASP1 might be expressed in dentate gyrus astrocytes. This is not surprising, given that radial NSCs give rise to astrocytes and that GFAP, vimentin, nestin and BLBP are all expressed in astrocytes as well. To then solidify our finding that NSC-6 does not label ANPs, we did a triple stain with NSC-6, GFAP and Sox2 antibodies. We confirmed that NSC-6 did not identify ANPs, which were only Sox2-positive (Fig. 5C). In addition, we utilized another transgenic mouse line in which nestin regulatory elements drive the expression of the cyan fluorescent protein (CFP) containing a signal for nuclear localization^35,36^. Since only the nuclei of NPCs can be visualized in the *Nestin*-CFPnuc mouse strain, NSCs and ANPs cannot be distinguished, unless an additional marker such as vimentin or GFAP is used. Our data employing NSC-6 immunolabeling in brain sections from *Nestin*-CFPnuc mice confirmed that NSC-6-labeled BASP1 is expressed only in NSCs within the SGZ neurogenic niche (Supplementary Fig. S3A). Immunostaining with the commercially available BASP-1 polyclonal antibodies did not yield any detectable staining (Supplementary Fig. S3B,C).

### The NSC-6 antibody localizes BASP1 to B and C cells in the postnatal SVZ and NSCs of the spinal cord

**Figure 6:**
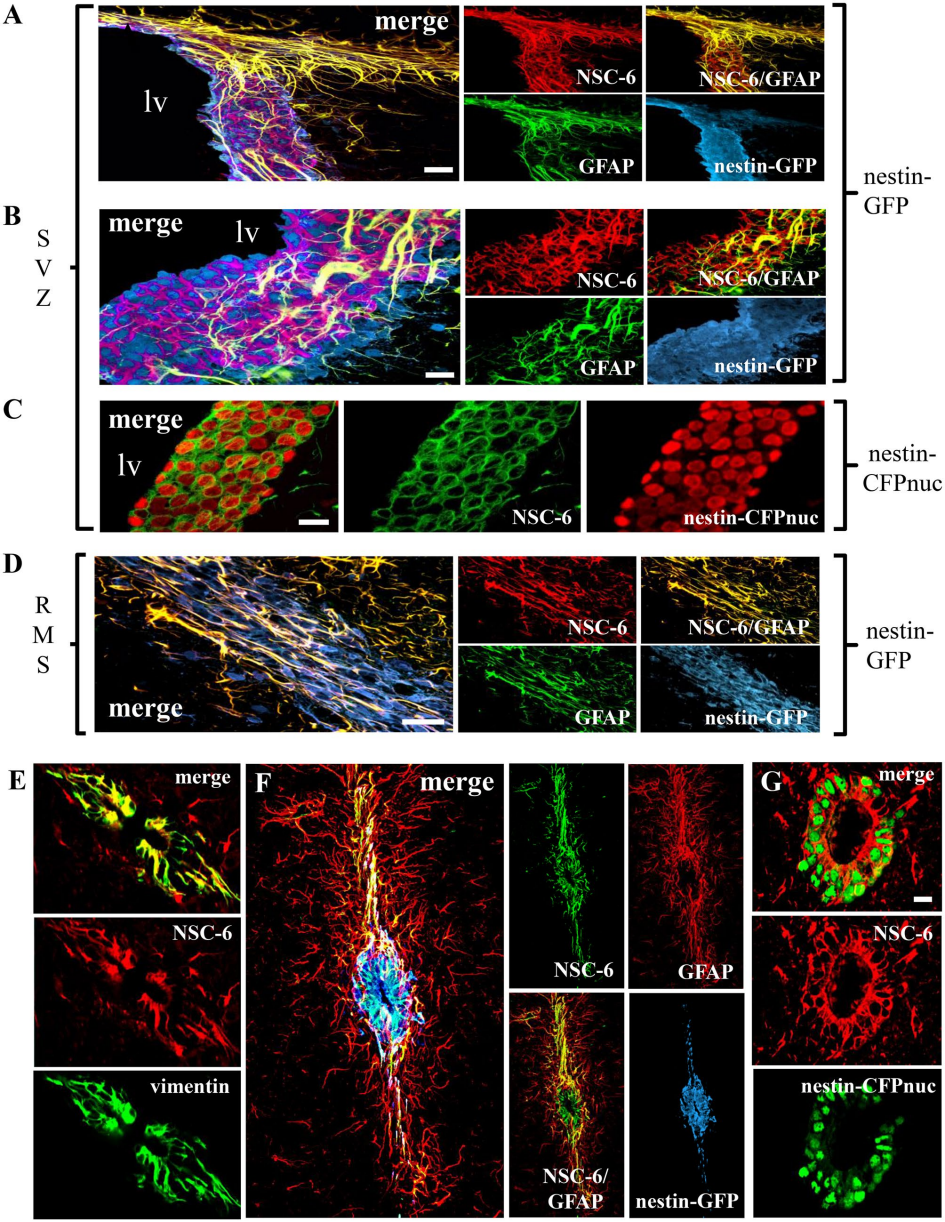
NSC-6 antibody immunolabels cells in the adult SVZ and RMS as well as around the spinal cord canal. (**A**) NSC-6 antibody immunostain co-localizes with GFAP and *Nestin*-GFP in the SVZ and the RMS (its origin is shown in the upper right corner of the images). (**B**) NSC-6 also immunolabels the cytoplasm of some GFAP-negative *Nestin*-GFP cells in the SVZ. (**C**) NSC-6 immunolabels the cytoplasm of SVZ neural stem and progenitor cells in *Nestin*-CFPnuc transgenic mice. (**D**) In the RMS, NSC-6 immunolabels cytoplasmic processes of GFAP-positive *Nestin-*GFP cells and co-localizes with GFAP in *Nestin*-GFP-negative cells. (**E**) NSC-6 co-localizes with vimentin in cells surrounding the central canal of the spinal cord. It also sparsely stains vimentin-negative cell processes in the neighboring parenchyma. (**F**) NSC-6 labels *Nestin*-GFP-positive GFAP-negative cells around the central canal, as well as *Nestin*-GFP-negative GFAP-positive cell processes in the surrounding parenchyma. (**G**) NSC-6 antibody labels the cytoplasm of the *Nestin*-CFPnuc cells surrounding the central canal in the *Nestin*-CFPnuc transgenic mice. Scale bars are 10 µm in (**A**,**C**–**E**,**G**); and 5 µm in (**B**), and 40 µm in (**F**).

In the SVZ, NSC-6 co-localized with GFP and GFAP in *Nestin*-GFP mice (Fig. 6A,B), therefore labeling B cells, the equivalent to NSCs in the hippocampus^40^. However, in the SVZ, NSC-6 also labeled C cells, the transient amplifying precursors (equivalent to hippocampal ANPs), which express nestin but not GFAP or vimentin. The NSC-6 Ab did not label neuroblasts, termed A cells^41,42^, in the SVZ, as there was no co-localization with PSA-NCAM (Fig. 5B). In the *Nestin*-CFPnuc transgenic mice, NSC-6 immunolabeling was present in the cytoplasm of all *Nestin*-CFPnuc expressing cells (B and C cells, Fig. 6C). In addition, the NSC-6 Ab labeled ependymal cells, which also express nestin, are adjacent to the cells bordering the ventricle, and are labeled with FoxJ1 (Supplementary Fig. S4).

In the RMS, A and C precursors migrate towards the olfactory bulb ensheathed by B cells^42^. NCS-6 immunolabeled astrocytic tubes that ensheath C cells (immunopositive for *Nestin*-GFP and GFAP) as they migrate toward the olfactory bulb and astrocytes (immunopositive for GFAP but not *Nestin*-GFP) located in the vicinity (Fig. 6D). *Nestin*-GFP-positive and GFAP-negative C cells were not immunolabeled with NSC-6 (Fig. 6D), which is in contrast to what we observed in the SVZ. Because of the dissimilarities between their respective immunolabeling patterns, we concluded that the NSC-6 Ab does not recognize nestin, GFAP, or vimentin and we validated this conclusion by biochemical analyses that showed distinct immunolabeling of the respective target proteins on immunoblots of whole mouse brain extracts (Fig. 8A–D). Taken together, NSC-6-labeled BASP1 expression is limited to B and C cells in the SVZ and only astrocytic tubes that ensheath C cells in the RMS.

To examine whether NSC-6 also labels NSCs localized around the central canal of the spinal cord, we stained spinal cord sections from wild-type (Fig. 6E), *Nestin*-GFP (Fig. 6F), and *Nestin*-CFPnuc transgenic mice (Fig. 6G). NSC-6 co-localized with vimentin, GFP in *Nestin*-GFP, and CFP in *Nestin*-CFPnuc mice. However, while co-localized with GFAP in *Nestin*-GFP cells, it also labeled some *Nestin*-GFP-positive but GFAP-negative cells, suggesting that in the spinal cord, it might label some neuroprogenitors and not only NSCs.

### NSC-6 antibody labels NSCs in human brain organoids

**Figure 7:**
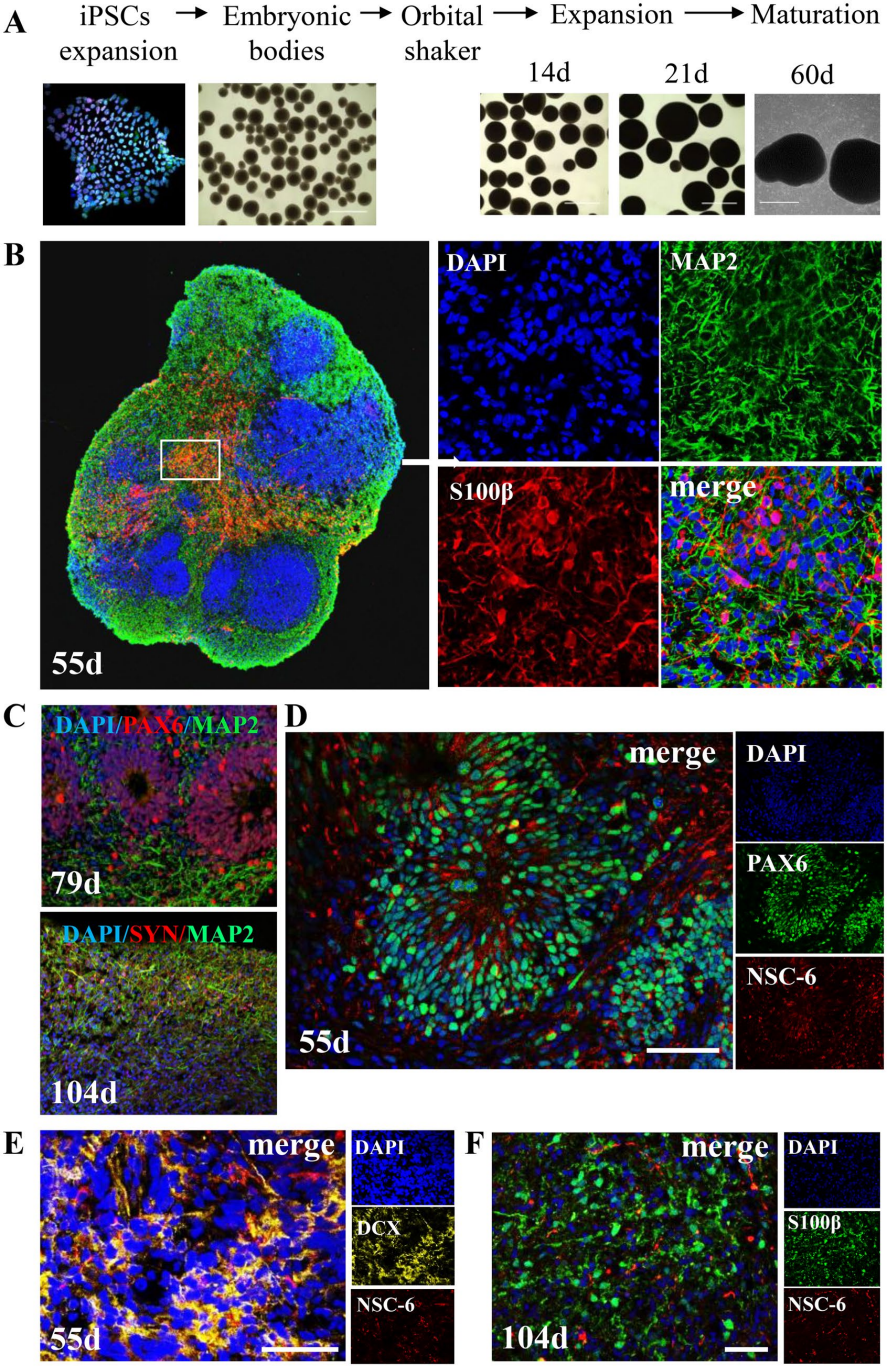
NSC-6 antibody labels NSCs and not neurons or astrocytes in human brain organoids. (**A**) Generation of forebrain organoids from human induced pluripotent cells (iPSCs) using modified Pasca method. The images show the key stages in organoid generation. (**B**) Cerebral forebrain organoids contain different cell populations at different stages of development, as shown by representative confocal micrographs of frozen organoid cross-sections. By 55d, the organoid contains ventricular-like zones where neural stem/progenitors reside, neurons (MAP2+), and astrocytes (S100β+). Inset is enlarged to show individual stains. (**C**) At 79d (upper panel), ventricular-like zones (PAX6+) are rare but still exist and neurons (MAP2+) are abundant. At 104d (lower panel), the organoid is populated with mature neurons with synaptic contacts (SYN1+). (**D**) Ventricular zone from a 55d organoid contains PAX6+ NSCs, many of which are labeled with NSC-6 antibody. NSC-6 antibody does not label immature DCX+ neurons in a 55d organoid (**E**) or S100β+ astrocytes in a 104d organoid (**F**). Scale bars 50 µm.

We used human iPSCs to generate brain organoids using modified Pasca protocol, a guided approach based on the supplementation of external factors to induce iPSCs to differentiate towards dorsal forebrain-like tissue^43^. We selected this method because it recapitulates with considerable accuracy the development of the human cortex in general and the organization of NSC zones in particular^44,45^. The key stages in organoid generation are shown (Fig. 7A). By day 55 (55d), the organoid contains ventricular-like zones where neural stem/progenitors reside; neurons (MAP2+), and astrocytes (S100β +) (Fig. 7B). At 79d, ventricular-like zones still exist (PAX6 ) and neurons are now abundant (MAP2+) (Fig. 7C, upper panel). By 104d, the organoid contains mature neurons with synaptic contacts as evidenced by SYN1 immunostaining (Fig. 7C, lower panel) and exhibits spontaneous electrical activity and action potentials (Supplementary Fig. S5). We examined the BASP1 expression in 55d organoids and observed that NSC-6 Ab strongly immunolabeled PAX6+ NSC subpopulation (Fig. 7D). In contrast, we did not detect such staining in immature DCX+ neurons in a 55d organoid (Fig. 7E) or mature S100β+ astrocytes in a 104d organoid (Fig. 7F). These data further solidify our findings in the mouse models, demonstrating that human NSCs are particularly enriched with BASP1 compared to other brain cells.

### NSC-6-labeled BASP1 is regulated temporally in the mammalian brain

**Figure 8:**
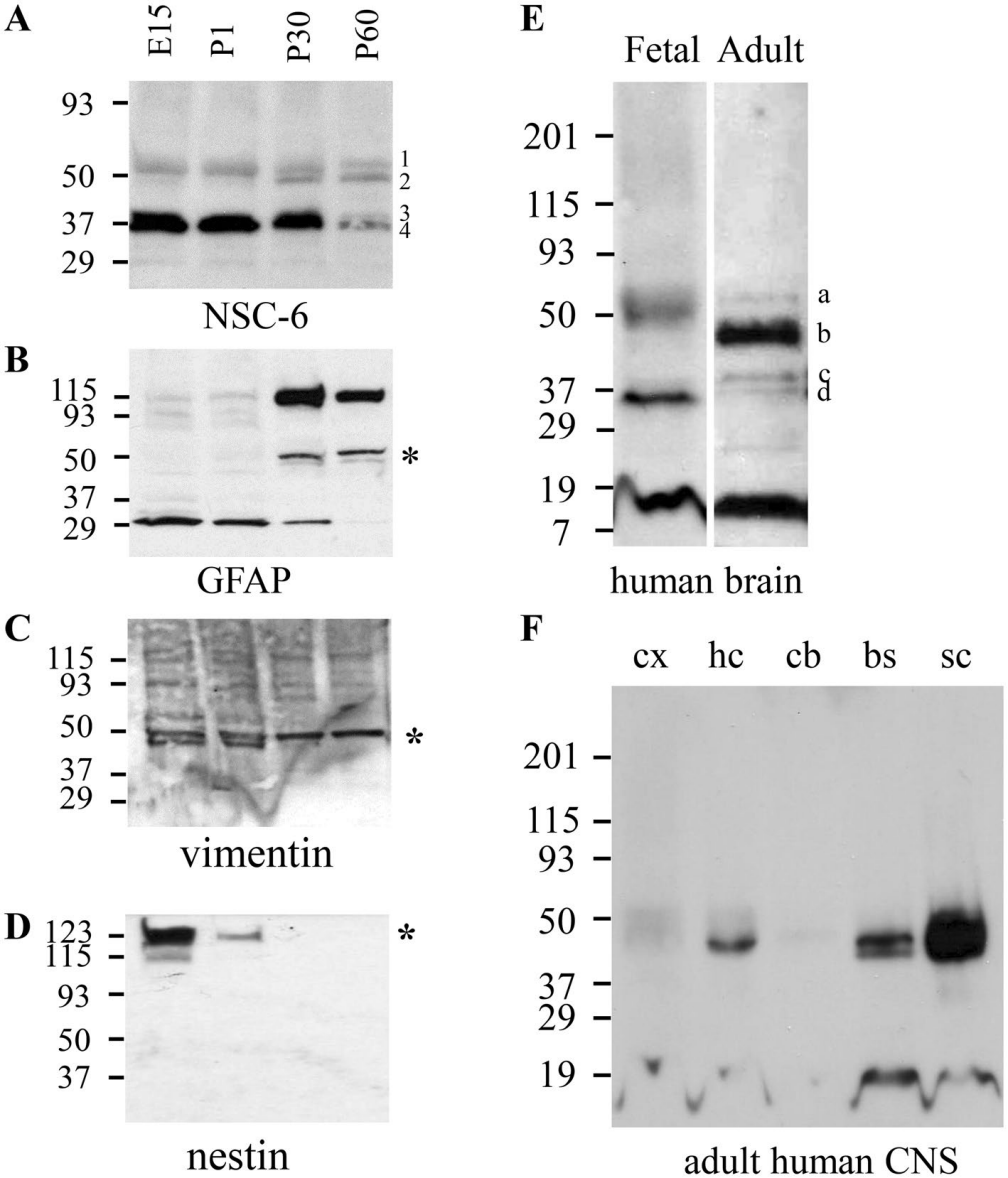
Immunoblot analysis of NSC-6 immunolabeling in developing mammalian brain. (**A**) NSC-6 immunoblotting of E15, P1, P30 and P60 mouse whole brain extracts. Four different bands (1–4) and with the respective M_r_ values of 51 kDa, 47 kDa, 38 kDa, and 35 kDa are recognized by the NSC-6 Ab. These distinct species changed through development and maturation. Immunoblots of the same samples probed with antibodies against GFAP (**B**), vimentin (**C**), and Nestin (**D**). The asterisks mark the bands corresponding to the expected M_r_ of these proteins. (**E**) NSC-6 immunoblotting of fetal and adult human brain. Note the distinct pattern of immunolabeling for the four bands a–d, with respective M_r_ values of 51 kDa, 45 kDa, 38 kDa, and 35 kDa, between fetal and adult brain. (**F**) NSC-6 immunoblotting of samples from human cortex (cx), hippocampus (hc), cerebellum (cb), brainstem (bs), and spinal cord (sc). Separate blots from different gels were cropped and separated by white space. Entire blots are not available.

We then examined temporal expression of the NSC-6-labeled BASP1. Biochemical analysis of the whole brain extracts from E15, P1, P30 and P60 mice by SDS-PAGE and immunoblotting revealed multiple polypeptide isoforms recognized by the NSC-6 Ab (Fig. 8A). The major isoforms observed in E15 and P1 mice correspond to relative electrophoretic mobilities (M_r_) of 35, 38, 47 and 51 kDa. In P30 mice, the 35 and 38 kDa isoforms were reduced relative to the 47 kDa isoform (Fig. 8A). Furthermore, in the P60 mouse brain this relative difference in isoform expression is even more apparent; there is a further reduction in the intensity of the 35 and 38 kDa bands while those corresponding to 47 and 51 kDa persist. These data suggest that BASP1 is modified during brain development such that isoforms exhibiting lower M_r_ values predominate early in development, while isoforms that exhibit higher M_r_ values become prevalent in adulthood. Positive controls included GFAP, vimentin, and nestin respectively (Fig. 8B–D). As expected, expression of GFAP was relatively low during development and increases with maturation (Fig. 8B). On the contrary, vimentin (Fig. 8C) and even more nestin (Fig. 8D) displayed robust expression during development with a rapid decline during maturation.

To then characterize BASP1 expression in the human brain, we compared fetal and adult human brain protein extracts by SDS-PAGE and NSC-6 immunoblot analysis. NSC-6 labeled a major band at 35 kDa and a minor band at 51 kDa in the fetal human brain (Fig. 8E). In the adult human brain, the major peptide recognized by NSC-6 migrated at 45 kDa (Fig. 8E) and relatively smaller quantities of the 51, 35 and 38 kDa isoforms were also detected. These results are generally consistent with those observed in the embryonic and adult mouse brain. Finally, we compared NSC-6 immunolabeling in samples prepared from specific regions of the human adult brain (Fig. 8F). Consistent with the data obtained in mice, our results show high BASP1 expression in the human hippocampus, the brainstem, and the spinal cord (Fig. 8F). As expected from the immunohistochemistry results reported above, relatively low levels of BASP1 expression were observed in the cortex and cerebellum.

## Discussion

In this study we aimed to generate mouse antibodies against epitopes found on NPCs. We isolated one antibody (NSC-6) and characterized it in detail. Mass spectrometry using human hippocampal tissue revealed the identity of the recognized antigen as BASP1, a signaling protein that plays a key role in neurite outgrowth and plasticity^14–19^, but here, we demonstrate that it might be utilized as a marker of NSCs in the adult brain.

Similar approaches to developing antibodies against mouse embryonic stem cells have been attempted in the past utilizing mice^46,47^ and rabbits^48^. Major drawbacks in mice include immune tolerance to mouse embryonic stem cell surface antigens leading to low antibody production, which could be overcome by immunizing rabbits instead. Regardless of the animal used as a host, a significant number of antibodies are typically generated against intracellular epitopes when animals are immunized with whole cells as was observed in our study.

We found that NSC-6-labeled BASP1 localizes to all radial glia at the E12 stage of brain development, while postnatally, it restricts to the neurogenic areas of the mouse brain but not the cortex. This expression pattern contrasts previous study using DAB-based immunolabeling for NAP-22 (BASP1 alias) in the adult rat brain, which demonstrated robust labeling of cerebral cortex^27^. While we do not know the basis of this difference in immunolabeling of cortex, possibilities include species variations between rat and mouse expression of BASP1, or differences in epitope recognition between the two antibodies used that could yield distinct patterns of immunoreactivity. Indeed, the two commercial BASP1 polyclonal antibodies did not immunolabel NSCs and in general, exhibited poor staining of the mouse brain tissue.

Given the reported data that BASP1 is expressed in neurons, we sought out to fully characterize the BASP1 expression in not only mouse brain, but also human brain organoids and human postmortem tissues. Our results suggest that BASP1 expression is restricted to NSCs and not present in neuroblasts, mature neurons, microglia, or oligodendrocytes. However, in both mouse neurogenic niches, NSC-6 antibody labeled sparse astrocytes. In contrast, in 3.5-month-old human brain organoids where astrocytes are fully mature, no NSC-6 immunolabeling was found. We can thus only speculate that in mouse astrocytes, BASP1 is a remnant in a subpopulation of astrocytes more closely related to the NSC lineage than those that do not express BASP1. Since BASP-1 is considered a signal processing protein playing key roles in synaptic plasticity and neurite outgrowth^14–19^, it is intriguing to speculate that BASP1 may play a role in maintaining NSC’s morphology as the radial processes are gone in ANPs or that it might serve as communication molecule between NSCs and granule cells in the dentate gyrus, mediating neuronal activity-dependent NSC activation.

Biochemical analysis of BASP1 using immunoblotting suggests that BASP1 migrates within a wide range of relative electrophoretic mobilities. Lower M_r_ species (35 and 38 kDa) predominate early during brain development while higher M_r_ species (47 and 51 kDa) are seen later in the adult brain. These data are consistent with the expression patterns of GAP-43, another growth-associated protein, in rat brain during development^20^. For example, GAP-43 mRNA expression is abundant throughout the developing human brain^49^ and declines during maturation^50^. Furthermore, prior studies have shown that GAP-43 migrates in an aberrant fashion on SDS gels, such that the protein’s M_r_ can vary between 43 and 57 kDa or even greater, depending on the acrylamide concentration^50,51^. The nature of these variations in M_r_ with developmental age is unclear. However, known natural N-terminal fragments of BASP1 known as BASP1-immunologically related proteins or BIRPs, do migrate on SDS gels as species with M_r_ values between 30 to 50 kDa^51^. These BIRP’s maintain N-terminal myristoylation but have varying C-terminal lengths, which accounts for their variations in electrophoretic mobility. The biological role of these fragments is unknown, but they are preserved in different tissues and species, suggestive of important functions. The presence of BIRPs may also explain why NSC-6 does not exhibit immunoreactivity in the cortex. At least six BIRPs have been described^52^ while our biochemical analyses detected only four major bands recognized by NSC-6. These findings may suggest that the NSC-6 Ab may recognize an epitope on BASP1 that is closer to the C-terminus, while other antibodies that detect more BIRPs and also exhibit immunolabeling in the cortex, may have epitopes localized closer to the N-terminus. Another antibody from our screen (NSC-32) only recognized the two higher M_r_ forms of BASP1 in adult and fetal brain tissues and did not exhibit immunolabeling in cortex either, suggesting that this epitope may be even further distal from the N-terminus, but not as proximal as NSC-6. Taken together, these data suggest that the lower forms of BASP1 predominate in fetal brain, while the higher M_r_ forms predominate in postnatal or adult tissue, not only in mice but also in humans. The significance of these tissue specific forms in unclear and should be explored in future studies.

In summary, we generated a mouse polyclonal antibody raised against mouse neurospheres that recognized BASP-1 by LC–MS/MS analysis of the immunoreactive proteins. Using this antibody, we discovered that BASP1 was expressed in the neurogenic regions of the mammalian brain including the hippocampus and SVZ as well as human brain organoids and postnatal human brain, robustly labeling NSCs. These findings suggest that BASP1 may serve as a marker of NSCs and play a role in brain development; however, further investigation will be necessary to determine its precise role.

## Materials and methods

Materials, data, and associated protocols will be made available to readers.

### Animals

All protocols and procedures for the use of animals were reviewed and approved by the Stony Brook University Animal Use and Care Committee; Cold Spring Harbor Laboratory Animal Use and Care Committee, and Baylor College of Medicine Animal Use and Care Committee. All experiments were performed in accordance with guidelines and regulations set forth by the Office of Research Compliance (ORC) at Stony Brook University, Cold Spring Harbor Laboratory, and Baylor College of Medicine.

### Embryonic neurosphere culture and hybridoma production

Whole brains from C57/BL6 embryonic day 12 (E12) mice were dissected, digested with collagenase (2 mg/ml, WORTHINGTON) for 2 h at 37 °C, filtered twice through 40 μm filters and plated at 3 × 10^5^ cells/10 ml of proliferation media containing Neurocult Basal Media (STEM CELL TECHNOLOGIES), 10% Proliferation Supplement (STEM CELL TECHNOLOGIES), 20 ng/ml EGF and FGF (SIGMA) and 1% antibiotic–antimycotic (GIBCO). Cells were cultured in 10 cm tissue culture dishes coated with 5% methylcellulose (SIGMA) at 37 °C under 5% CO_2_ with EGF and FGF supplementation every 2 to 3 days. Neurospheres were visible after 7–10 days in culture.

For hybridoma production, eight-week-old female BALB/C mice (N = 3) were injected intraperitoneally with 1 × 10^6^ cells from cultured neurospheres in PBS seven times in total (once every 2 weeks for 2 months and once a month for the last three months). Neurospheres were mechanically dissociated to single cells prior to injection. Hybridoma cells were produced by fusing spleen cells with NS-1 myeloma cells at a ratio of 1:10^53^. The fusion mixture was plated onto 30 × 96-well plates. Hybridomas were selected for growth in media containing 20% FBS + DMEM + Pen + Strep + 1× OPI (oxaloacetate, pyruvate and insulin) and azaserine + hypoxanthine, aminopterin, thymidine (HAT medium). Ten to fourteen days after the fusion, the conditioned medium from the 96 well plates (tissue culture supernatants) were screened by ELISA against cultured neurospheres. 1648 clones were screened and 39 exhibited immunoreactivities against cultured neurospheres and were subsequently subcloned and named Neural Stem Cell 1 to 39 (NSC 1–39).

### Two-dimensional electrophoresis

Two-dimensional electrophoresis (2-DE) was conducted using human hippocampal lysates described above^54^. Briefly, IPG strips (11 cm, pH range 3–10, BIO-RAD, cat # 163-2014) were passively equilibrated under mineral oil for 18 h at 23 °C with 90 µg of solubilized protein in 200 µl ST_50_. The IPG strips were subsequently washed by dipping into deionized H_2_O and blotted with filter paper. The strips were transferred to a Protean IEF (BIO-RAD) focusing tray and laid gel side down across wet paper wick-covered electrodes and covered with mineral oil. The strips were focused in a Bio-Rad Protean IEF Cell at 20 °C, rapid ramp 0–250 V, 15 min; rapid ramp 250–8000 V 2 h; hold 8000 V to a total of 35,000 Vh. The strips were subsequently removed from the focusing tray, blotted with moist filter paper and typically placed gel side up in channels of a clean rehydration/ equilibration tray, covered and stored at − 80 °C. The thawed strips were equilibrated at 23 °C for 10 min in equilibration buffer (EB) composed of 6 M urea, 50 mM Tris–HCl, pH 8.8, 2% SDS, 20% glycerol with 2% (w/v) DTT. The EB was decanted off and the strip equilibrated (10 min, 23 °C) in EB with 2.5% (w/v) iodoacetamide. The strips were transferred to IPG wells of pre-cast Criterion 8–16% polyacrylamide gels (BIO-RAD, cat# 345-0105) and overlaid with agarose. The gels were resolved under constant 200 V, 55 min.

### Spot excision and trypsin digestion

Excision and digestion protocols were performed by the Proteomics Center at Stony Brook and adapted from^55^. The film (NSC-6 immunoblot) was carefully overlaid and aligned with the original blot, which was sandwiched in a clear plastic envelope. Using a new 22-gauge needle for each spot, the outer edges of the spots were demarcated by a series of needle punctures passed through the film and the blot. The outlined spots were excised with a clean razor blade and transferred to 1.5 ml polypropylene centrifuge tubes. The membrane was dissolved in 500 μl of methanol and the methanol subsequently removed under a stream of nitrogen or in a Speed-Vac (SAVANT) apparatus. The resultant residue was digested in the presence of acetonitrile. The residue received 5 µl of 5× Invitrosol (INVITROGEN, cat# MS10007)) and 20 µl 25 mM NH_4_HCO_3_, and was heated at 60 °C, 5 min. The sample was cooled to room temperature and diluted with 100 µl of acetonitrile. The sample was briefly vortexed, centrifuged and then sonicated in a bath sonicator for 2 h, maintaining bath temperature near room temperature. At the end of this procedure, the nitrocellulose was finely dispersed. The suspension received 20 µl of trypsin (PROMEGA Gold, Mass Spectrometry Grade, cat# V5280) at 20 ng/µl, and was digested overnight at 37 °C. The incubate was centrifuged for 5 min at 16,000×*g*, and the supernatant transferred to a clean 500 µl polypropylene tube. The original tube was rinsed with 50 µl of 2% acetonitrile, 0.1% formic acid (buffer A), centrifuged and the rinse combined with the original supernatant. The combined solutions were evaporated in a Speed-Vac apparatus to a final volume of 10 µl. The samples were purified using a C_18_ Zip-Tip (MILLIPORE, cat# ZTC18S096) column and evaporated to 5 µl in a Speed-Vac. The final product received 1 µl formic acid and was diluted to a volume of 15 µl with buffer A.

We analyzed the peptide mixture using automated microcaplillary liquid chromatography-tandem mass spectrometry as reported by Link AJ, 1999^56^. First, we pulled fused-silica capillaries (100 μm i.d.) using a P-2000 CO2 laser puller (Sutter Instruments, Novato, CA). These had a 5 μm i.d. tip and were filled up 10 cm length with 5 μm Magic C18 (AGILENT, Santa Clara, CA). We placed this column in-line with a Dionex Ultimate 3000 that has an autosampler. We used buffer A to equilibrate the column and the autosampler to load the peptide mixture onto the column. The running parameters were as follows: HPLC pump flow 100 μl/min; the flow rate to the electrospray tip ~ 200–300 nl/min. The gradient between Buffer A and Buffer B (90% ACN, 0.1% FA) enabled HPLC separation. After peptide loading, we held the HPLC gradient constant at 100% buffer A for 5 min, followed by 5% buffer B to 40% buffer B for 30 min. Then, we switched the gradient from 40 to 80% buffer B over 5 min and held it constant for 3 min. Finally, we changed it from 80% buffer B to 100% buffer A over 1 min, and held it constant at 100% buffer A for an additional 15 min. 1.8 kV distal voltage electrosprayed the eluted peptides directly into a Thermo Fisher Scientific LTQ XL ion-trap mass spectrometer equipped with a nanoLC electrospray ionization source (THERMO-FINNIGAN, San Jose, CA). We recorded full mass (MS/MS) peptide spectra over a 400–2000 m/*z* range, followed by five tandem-mass (MS/MS) events sequentially generated in a data-dependent manner on the first, second, third, fourth, and fifth most intense ions selected from the full MS spectrum (at 35% collision energy). We used the Xcalibur data system (THERMO-FINNIGAN) to control the mass spectrometer scan functions and HPLC solvent gradients.

MS/MS spectra were extracted from the RAW file with Readw.exe (http://sourceforge.net/projects/sashimi). The resulting mzXML file contains all the data for all MS/MS spectra and can be read by the subsequent analysis software. The MS/MS data was searched with Inspect^57^ against a human IPI database with optional modifications: + 16 on Methionine, + 57 on Cysteine, + 80 on Threonine, Serine and Tyrosine. Only peptides with at least a p-value of 0.025 were analyzed further. Common contaminants (e.g., keratins) were removed from the returned data set. Proteins identified by at least three distinct peptides within a sample were considered valid; when sample signal was very weak, two distinct peptides were accepted for a valid identification. Further validation of sequences of interest was obtained by manual inspection of the MS/MS spectra.

### Flow cytometry

Plated human iPSC-derived NPCs (hNPCs) were harvested using Accutase cell dissociation solution (GIBCO), washed in ice-cold flow buffer (DPBS, 2% fetal bovine serum, 2 mM EDTA). In each condition, 1 × 10^6^ cells were incubated with the NSC-6 antibody for 45 min on ice (1:20; optimal concentration determined by previous titration experiments). The cells were then washed twice in ice-cold flow buffer with 2% normal donkey serum added, then stained in the same buffer with Alexa Fluor 405 (1:200 donkey anti-mouse IgG H&L; ABCAM 175659) secondary antibody for 45 min on ice. Stained cells were washed twice in ice-cold flow buffer then resuspended in a viability dye solution (20 mM Helix NP NIR, BIOLEGEND, 425301 in flow buffer) and immediately analyzed by flow cytometry using an LSR Fortessa (BECTON DICKINSON) with acquisition of AF405 on V450 and Helix-NIR on R670. Final data analyses were performed using FlowJo software (TREE STAR INC.). To determine hNPC NSC-6 positivity, 250,000 live cell events were acquired. NSC-6 positive signal was set by gating for single viable cells and above background, the latter determined using the signals obtained from unstained hNPCs and hNPCs stained with the secondary antibody only.

### Neurosphere culture from adult brain SVZ

SVZ from the 1-month-old C57Bl6 mice was dissected out in HBSS and tissue was minced in fine pieces. The minced tissue was transferred to 1.25 ml of 0.1% Trypsin and incubated at 37 °C for 7 min with intermittent agitation every 2 min by hand. 3 ml of Trypsin inhibitor (from glycine max) was added and gently mixed and the tissue suspension was passed through 70 μm pore size filter followed by centrifuge at 700 rpm for 5 min. The supernatant was removed and the pellet was resuspended in the NSC culture media (DMEM: F12 media 46.95 ml, N2 0.5 ml, B27 1 ml, PSG 0.5 ml, 1 M KCl, 2mg/ml Heparin 0.05 ml, bFGF 10ng/ml and EGF-2 200ng/ml). Growth factors were added every day for the first week and after passaging, growth factors were added every other day. Neurospheres are visible after 6–7 days and were collected 2 weeks after plating.

### mRNA expression by quantitative real time PCR assay

Total RNA was extracted from the cultured neurospheres and tissue obtained from the 3-month-old C57Bl6 mouse liver by Aurum Total RNA mini kit (BIO RAD) according to manufacturer’s instructions. Equal mRNA amount was used for cDNA synthesis by High Capacity cDNA Reverse Transcription kit (APPLIED BIOSYSTEM). 40 ng of cDNA was amplified in 20 μl of reaction mixture containing 5 μl of 2X Power SYBR green PCR Master Mix (APPLIED BIOSYSTEM), 0.5 μl forward primer, and 0.5 μl reverse primers. Forward Primer sequence was 5′GCGAGGCCAAAAAGACTGAG 3′ and Reverse primer sequence was 5′CCGCGCTGCTAGGTTTAGAG3′. All reactions were performed in triplicate on Bio Rad CFX Real Time PCR system. Amplification conditions comprised of initial holding stage of 95 °C for 10 min then denaturation at 95 °C for 15 s, further annealing and elongation at 60 °C for 1 min for 40 cycles, followed by melt curve stage for each gene of interest. GAPDH was used as an endogenous control. The value of each Ct was normalized by Ct value of GAPDH. The relative gene expression was defined as 2^-ΔΔCt^ and final gene expression was represented as 2^−ΔΔCt±SEM^. Two-tailed unpaired T-test with Welch correction was used to analyze the difference between BASP1 expression in different tissue type.

### Human brain organoids and hNPCs

Human iPSCs were obtained from the Human Neuronal Differentiation Core at the Neurological Research Institute that derived them from a healthy adult male according to an IRB-approved protocol and established reprogramming methods. They were maintained at undifferentiated state by growing on matrigel (CORNING) in Essential 8 Flex Medium (GIBCO). Human brain organoids and 2D culture of hNPCs were generated according to modified protocols by Palm et al.^58^ and Sloan et al.^59^ respectively. Specifically, iPSCs colonies were first dissociated into single cells (D0) with Accutase (SIGMA-ALDRICH) and 1.5 million cells seeded per well of an AggreWell800 plate (STEM CELL TECHNOLOGIES) in a medium containing KnockOut DMEM, 20% KnockOut Serum Replacement, 1% penicillin and streptomycin (P/S) solution, 0.5X GlutaMAX, 1× MEM Non-Essential Amino Acids (all form GIBCO), 5 µM Dorsomorphin (PEPROTECH), 10 µM SB431542 (PEPROTECH), 100 µM 2-Mercaptoethanol (SIGMA), and 10 µM Y-27632 (TOCRIS). On D1, 75% of the medium was replaced with the same medium but without Y-27632. On D2 and D3, half and 75% of the medium was replaced, with N2B27 medium containing DMEM/F-12 (with glutamine) and Neurobasal Medium at 1:1 ratio, 0.5X GlutaMAX, 0.5X-N2 supplement, 0.5X-B27 (-A) supplement, 1X P/S (all from GIBCO), 5 µM Dorsomorphin, and 10 µM SB431542, respectively. On D5 and D6, 75% of the medium was replaced with a fresh N2B27 medium supplemented with 150 µM l-Ascorbic acid (AA) (SIGMA-ALDRICH). On D8, the aggregates were collected from the AggreWell800 microwells and transferred to grow in N2B27 medium, supplemented with 150 µM AA and 20 ng/ml bFGF (PEPROTECH), in non-treated tissue culture plates on an orbital shaker (70 rpm). After additional 2 weeks of growth during which the cell aggregates consist of mainly NPCs, they were either treated to form 2D cultures of hNPCs or to form 3D organoids. To generate and maintain *2D hNPC cultures*, the cell aggregates were first seeded on Poly-l-Ornithine (SIGMA)/Laminin (CORNING) coated plates in a medium containing DMEM/F12 (with Glutamine), 1X-N2 and 1X-B27(-A) supplements, 20 ng/ml bFGF, and 1% P/S. Five days later, and every 3–4 days thereafter, the culture was passaged as single cells using Accutase and grown on Poly- l -Ornithine/Laminin in NPC medium containing DMEM/F12 (with glutamine), 0.5X GlutaMAX, 1X MEM Non-Essential Amino Acids, 1% P/S, X1 N2 and X1 B27 with vitamin A (GIBCO) supplements, 40 ng/ml bFGF, 40 ng/ml EGF (PEPROTECH), and 1.5 ng/ml LIF (PEPROTECH)^52^. To induce maturation of the *3D organoids*, the N2B27 medium was supplemented with 20 ng/ml BDNF and 20 ng/ml NT3 growth factors (PEPROTECH). 60 days from the start of iPSC differentiation, BDNF and NT3 were omitted from the medium and organoids allowed to expand and mature.

### Immunohistochemistry

#### Mouse models

NSC-6 immunolabeling was examined in samples from wild-type C57/BL6, *Nestin*-GFP, and *Nestin*-CFPnuc mice. For experiments on *embryos*, E12 embryos were dissected and quickly decapitated. The whole heads were fixed in 4% formaldehyde in phosphate-buffered saline (PBS) for 24 h. The brains were dissected out and sectioned with a Vibratome 1500 (VIBRATOME). Immunolabeling was carried out following a standard procedure with some modifications^52^: the sections were incubated with blocking and permeabilization solution (PBS containing 0.2% Triton-100X and 3% BSA) for 1 h at room temperature, and then incubated overnight with the primary antibodies (diluted in the same solution) at 4 °C. For experiments on *adult mice*, these were all between 8 and 16 weeks of age^5,60,61^. They were transcardially perfused with 30 ml of PBS followed by 30 ml of 4% (w/v) formaldehyde (prepared fresh from paraformaldehyde) in PBS, pH 7.4. The brains were removed, cut longitudinally into two hemispheres and postfixed with the same fixative for 3 h at room temperature, then transferred to PBS and kept at 4 °C. Serial 40 μm thick sagittal sections were cut using a Vibratome 1500 (VIBRATOME, St Louis, MO).

The following primary antibodies were used: chicken anti-GFP (AVES LABORATORIES, Tigard, OR) at 1:500 dilution; chicken anti-vimentin (CHEMICON INTERNATIONAL, Temecula, CA) at 1:1000; goat anti-DCX (SANTA CRUZ BIOTECHNOLOGY, Santa Cruz, CA) at 1:500; rabbit anti-BLBP (CHEMICON) at 1: 500; rabbit anti-GFAP (SIGMA) at 1:1000; rat anti-GFAP (Cat# 13-0300, THERMO FISHER SCIENTIFIC, Waltham, MA) at 1:1000; rabbit anti-Iba-1 (UPSTATE BIOTECHNOLOGY, Lake Placid, NY) at 1:2000; rabbit anti-MBP (CHEMICON); rabbit anti-NeuN (BOSTER BIOLOGICAL Technology), rabbit anti-Sox2 (ab97959, ABCAM PLC), rabbit anti-NG2 (CHEMICON) at 1:200; rabbit anti-Prox-1 (CHEMICON) at 1:100; AlexaFluor 488 goat anti-chicken (MOLECULAR PROBES, Willow Creek Road, Eugene, OR) at 1:500; AlexaFluor 568 goat anti-mouse (MOLECULAR PROBES) at 1:500; AlexaFluor 488 goat anti-mouse (MOLECULAR PROBES) at 1:500; Cy5 goat anti-rabbit (JACKSON IMMUNORESEARCH, West Grove, PA) at 1:500; and Texas Red donkey anti-chicken (JACKSON IMMUNORESEARCH) at 1:500. Human FoxJ1 Antibody (AF3619) BIO-TECHNE R&D Systems, S.L.U. Dilution: 1/250 (or 1.14 μg/ml). NSC-6 antibody was used at 1:20. Commercially available BASP-1 antibodies used were BIOSS (bs-8662R) and THERMO FISHER SCIENTIFIC (PA-78320). The CFP signal from the *Nestin*-CFPnuc transgenic mice was detected with an antibody against GFP for enhancement and better visualization. For *immunofluorescence labeling*, after thorough washing with PBS, the sections were incubated with fluorochrome-conjugated secondary antibodies diluted in the permeabilization and blocking solution for 1 h at room temperature. After washing with PBS, the sections were mounted on gelatin-coated slides with DakoCytomation Fluorescent Mounting Medium (DAKOCYTOMATION, Carpinteria, CA). For *DAB staining*, after thorough washing with PBS, the sections were incubated with biotinylated secondary antibodies in PBS for 1 h at room temperature. After washing, the sections were incubated with peroxidase-linked ABC (Vector Labs). The peroxidase activity was demonstrated using the Fast 3, 3′-diaminobenzidine tetrahydrochloride with metal enhancer tablet set (SIGMA-ALDRICH, St Louis, MO).

#### Human models

For experiments on *2D hNPCs*, cells were thoroughly washed and then fixed for 15 min with 4% paraformaldehyde and 1.1–1.6% methanol in PBS. They were then washed with PBS and stored at 4 °C. Prior to immunostaining, they were permeabilized with 0.1% Triton X-100 in PBS for 15 min, and blocked with a blocking solution containing 5% normal donkey serum, 2% BSA, and 0.1% Triton X-100 in PBS for 1––2 h at room temperature. They were then incubated overnight at 4 °C with primary antibodies diluted in the blocking solution. For experiments on *brain organoids*, they were washed with PBS and fixed for 4 h (55d organoids) or 24 h (104d organoids) at 4 °C with 4% Paraformaldehyde and 1.1–1.6% methanol in PBS. The organoids were washed with PBS and transferred into 30% sucrose in PBS solution for 2 days at 4 °C. They were embedded in OCT (TISSUE TEK) and sectioned into 14 μm sections by a cryostat. Prior to immunostaining, organoid sections were permeabilized with 0.1% Triton X-100 in PBS for 15 min and blocked with a blocking solution containing 5% normal donkey serum, 2% BSA, and 0.1% Triton X-100 in PBS for 1–2 h at room temperature. They were then incubated overnight at 4 °C with primary antibodies diluted in the blocking solution. Primary antibodies used were: rabbit anti- PAX6 (GENETEX) at 1:130 dilution ratio, mouse anti-Nestin (MILLIPORE-SIGMA) at 1:200, goat anti-Doublecortin (DCX, SANTA CRUZ BIOTECHNOLOGY) at 1:200, rabbit anti-S100 (DAKO) at 1:300, and NSC-6 antibody at 1:20. After washing with 0.1% Triton X-100 in PBS, they were incubated with compatible fluorescence secondary antibodies for 2 h at room temperature. Secondary antibodies used (all diluted 1:500 in blocking buffer) were: AlexaFluor 488 donkey anti-rabbit (MOLECULAR PROBES), AlexaFluor 647 donkey anti-rabbit (MOLECULAR PROBES), AlexaFluor 647 donkey anti-mouse (MOLECULAR PROBES), and Rhodamine Red-X AffiniPure donkey Anti-goat (JACKSON IMMUNORESEARCH). Cells were washed with 0.1% Triton X-100 in PBS and nuclei stained with DAPI (4′, 6-diamidino-2-phenylindole) in PBS for 10 min at room temperature. Cells were then washed with PBS and slides were mounted using ProLong Diamond Antifade Mountant (INVITROGEN).

### Microscopy

All imaging of mouse sections was acquired using a laser scanning confocal microscope LSM 510 (CARL ZEISS, Thornwood, NY) and the corresponding manufacturer’s software. The signal from each fluorochrome was collected sequentially (multi-track setting). Control sections stained with single fluorochromes confirmed full antibody penetration and lack of spectral overlap between different channels. All images shown correspond to projections of 10 to 15 μm z-stacks. All fluorescence immunolabeling images for human iPSC-derived cultures were collected using Leica SP8X confocal microscope, and the corresponding manufacturer’s software. DAB-based immunolabeling images were taken using and upright bright field microscope (CARL ZEISS).

### Immunoblot analysis

Immunoblot analysis was carried out using crude E12 to P30 C57/BL6 mouse brain extracts and analyzed by SDS-PAGE. The preparation of crude mouse brain membranes and their subsequent analysis by immunoblots were performed as described^54^. Protein quantities were determined using the BCA method (PIERCE). Human Hippocampus Tissue Lysate (Cat. No. XBL-10110) was obtained from PROSCI. INCORPORATED. The lysate proteins were obtained by TCA precipitation using the BIO-RAD ReadyPrep 2-D Cleanup Kit (cat# 163-2130) according to the kit protocol. The resultant precipitated protein was dissolved in first dimension rehydration buffer (ST_50_) consisting of 7 M urea, 2 M thiourea, 4% CHAPS, 0.2% BIO-RAD Biolytes (3–10) and 50 mM dithiothreitol (DTT). The method yielded approximately 80%, by mass, of the lysate proteins.

## Supplementary Information

Supplementary Information.
