# Author Correction: BASP1 labels neural stem cells in the neurogenic niches of mammalian brain

**DOI:** 10.1038/s41598-021-99309-6

**Published:** 2021-10-04

**Authors:** Louis N. Manganas, Irene Durá, Sivan Osenberg, Fatih Semerci, Mehmet Tosun, Rachana Mishra, Luke Parkitny, Juan M. Encinas, Mirjana Maletic‑Savatic

**Affiliations:** 1 Department of Neurology, Stony Brook University Medical Center, Stony Brook, NY USA; 2 Achucarro Basque Center for Neuroscience, Leioa, Spain; 3 The Basque Foundation for Science, IKERBASQUE, Bilbao, Spain; 4 Department of Neuroscience, University of the Basque Country (UPV/EHU), Leioa, Spain; 5 Departments of Pediatrics, Neurology and Neuroscience, Baylor College of Medicine, Houston, TX USA; 6 Jan and Dan Duncan Neurological Research Institute at Texas Children’s Hospital, Houston, TX USA; 7 Department of Neurology, Stony Brook University Medical Center, Health Sciences Center T‑12, room 020, Stony Brook, NY 11794 USA; 8 Departments of Pediatrics, Neurology, and Neuroscience, Baylor College of Medicine, Jan and Dan Duncan Neurological Research Institute at Texas Children Hospital, 1250 Moursund St., Rm 1250, Houston, TX 77030 USA

Correction to: *Scientific Reports* 10.1038/s41598-021-85129-1, published online 10 March 2021

The original version of this Article contained errors.

Author Fatih Semerci was incorrectly given as Faith Semerci.

In addition, in the Introduction,

“The NSC-6 Ab, which produced the most robust staining of NPCs, corresponded to Brain-Associated Signal Protein 1 (BASP1), a protein not previously described in NPCs.”

now reads:

“The NSC-6 Ab, which produced the most robust staining of NPCs, corresponded to Brain-Abundant, membrane-attached Signal Protein 1 (BASP1), a protein not previously described in NPCs.”

In the Results section, under subheading “NSC-6 stains mouse and human NPCs”,

“In addition, we derived human neuroprogenitor cells (hNPCs) from inducible pluripotent stem cells (iPSCs) obtained from a healthy adult and stained them with NSC-6.”

now reads:

“In addition, we derived human neuroprogenitor cells (hNPCs) from induced pluripotent stem cells (iPSCs) obtained from a healthy adult and stained them with NSC-6.”

Under the subheading “NSC-6-labeled BASP1 is regulated temporally in the mammalian brain”,

“Consistent with the data obtained in mice, our results show high BASP1 expression in the hippocampus, the brainstem, and the spinal cord (Fig. 8F).”

now reads:

“Consistent with the data obtained in mice, our results show high BASP1 expression in the human hippocampus, the brainstem, and the spinal cord (Fig. 8F).”

In the Materials and methods section, under subheading “Embryonic neurosphere culture and hybridoma production”,

“Whole brains from C57/BL6 embryonic day 12 (E12) mice were dissected, digested with collagenase (2 mg/ml, WORTHINGTON) for 2 h at 37 °C, filtered twice through 0.4 μm filters and plated at 3 × 10^5^ cells/10 ml of proliferation media containing Neurocult Basal Media (STEM CELL TECHNOLOGIES), 10% Proliferation Supplement (STEM CELL TECHNOLOGIES), 20 ng/ml EGF and FGF (SIGMA) and 1% antibiotic–antimycotic (GIBCO).”

now reads:

“Whole brains from C57/BL6 embryonic day 12 (E12) mice were dissected, digested with collagenase (2 mg/ml, WORTHINGTON) for 2 h at 37 °C, filtered twice through 40 μm filters and plated at 3 × 10^5^ cells/10 ml of proliferation media containing Neurocult Basal Media (STEM CELL TECHNOLOGIES), 10% Proliferation Supplement (STEM CELL TECHNOLOGIES), 20 ng/ml EGF and FGF (SIGMA) and 1% antibiotic–antimycotic (GIBCO).”

Under subheading “Two-dimensional electrophoresis”,

“Briefly, IPG strips (11 cm, pI range 3–10, BIO-RAD, cat # 163-2014) were passively equilibrated under mineral oil for 18 h at 23 °C with 90 µg of solubilized protein in 200 µl ST_50_.”

now reads:

“Briefly, IPG strips (11 cm, pH range 3–10, BIO-RAD, cat # 163-2014) were passively equilibrated under mineral oil for 18 h at 23 °C with 90 µg of solubilized protein in 200 µl ST_50_.”

Under the subheading “Neurosphere culture from adult brain SVZ”,

“3 ml of Trypsin inhibitor (from glycine max) was added and gently mixed and the tissue suspension was passed through 0.7 μm pore size filter followed by centrifuge at 700 rpm for 5 min. The supernatant was removed and the pellet was resuspended in the NSC culture media (DMEM: F12 media 46.95 ml, N2 0.5 ml, B27 1 ml, PSG 0.5 ml, 1 M KCl 1 ml, Heparin 0.05 ml, bFGF 1 μl/10 ml and EGF-2 1 μl/10 ml).”

now reads:

“3 ml of Trypsin inhibitor (from glycine max) was added and gently mixed and the tissue suspension was passed through 70 μm pore size filter followed by centrifuge at 700 rpm for 5 min. The supernatant was removed and the pellet was resuspended in the NSC culture media (DMEM: F12 media 46.95 ml, N2 0.5 ml, B27 1 ml, PSG 0.5 ml, 1 M KCl, 2mg/ml Heparin 0.05 ml, bFGF 10ng/ml and EGF-2 200ng/ml).”

Under the subheading, “Human brain organoids and hNPCs”,

“Specifically, iPSCs colonies were first dissociated into single cells (D0) with Accutase (SIGMA-ALDRICH) and 1.5 million cells seeded per well of an AggreWell00 plate (STEM CELL TECHNOLOGIES) in a medium containing KnockOut DMEM, 20% KnockOut Serum Replacement, 1% penicillin and streptomycin (P/S) solution, 0.5X GlutaMAX, 1× MEM Non-Essential Amino Acids (all form GIBCO), 5 µM Dorsomorphin (PEPROTECH), 10 µM SB431542 (PEPROTECH), 100 µM 2-Mercaptoethanol (SIGMA), and 10 µM Y-27632 (TOCRIS).”

now reads:

“Specifically, iPSCs colonies were first dissociated into single cells (D0) with Accutase (SIGMA-ALDRICH) and 1.5 million cells seeded per well of an AggreWell800 plate (STEM CELL TECHNOLOGIES) in a medium containing KnockOut DMEM, 20% KnockOut Serum Replacement, 1% penicillin and streptomycin (P/S) solution, 0.5X GlutaMAX, 1× MEM Non-Essential Amino Acids (all form GIBCO), 5 µM Dorsomorphin (PEPROTECH), 10 µM SB431542 (PEPROTECH), 100 µM 2-Mercaptoethanol (SIGMA), and 10 µM Y-27632 (TOCRIS).”

Lastly, Reference 11 was incorrectly given as:

Semerci, F. & Maleic-Savatic, M. Transgenic mouse models for studying adult neurogenesis. *Front. Biol. (Beijing)* **11**, 151–167 (2016).

The correct reference is listed below:

Semerci, F. & Maletic-Savatic, M. Transgenic mouse models for studying adult neurogenesis. *Front. Biol. (Beijing)* **11**, 151–167 (2016).

The original Article has been corrected.

