# Supplementary material for: BASP1 labels neural stem cells in the neurogenic niches of mammalian brain
Source: Sci Rep. 2021 Mar 10;11:5546. doi: 10.1038/s41598-021-85129-1 (PMC7970918; doi:10.1038/s41598-021-85129-1)
Supplement: Supplementary file 1 — Supplementary Information. [file 41598_2021_85129_MOESM1_ESM.pdf]

Running title: BASP1 and neurogenesis

BASP1 Labels Neural Stem Cells in the Neurogenic Niches of Mammalian Brain

Louis N. Manganas[a#], Irene Durá[b, d], Sivan Osenberg[e,f], Fatih Semerci[e,f], Mehmet Tosun[e,f], Rachana Mishra[e,f], Luke Parkitny[e,f], Juan M. Encinas[b, c, d] and Mirjana Maletic- Savatic[a,e,f#]

[a] Department of Neurology, Stony Brook University Medical Center, Stony Brook, New York, United States of America

[b] Achucarro Basque Center for Neuroscience, Spain

[c] IKERBASQUE, the Basque Foundation for Science, Spain

[d] Department of Neuroscience, University of the Basque Country (UPV/EHU), Spain

[e] Departments of Pediatrics, Neurology and Neuroscience, Baylor College of Medicine, Houston, Texas, United States of America

[f] Jan and Dan Duncan Neurological Research Institute at Texas Children's Hospital, Houston, Texas, United States of America

# Corresponding authors:

Louis N. Manganas, MD, PhD
Associate Professor, Department of Neurology
Stony Brook University Medical Center
Health Sciences Center T-12, room 020
Stony Brook, NY 11794
Tel 631 444 2599 Fax 631 444 1474 email: louis.manganas@stonybrookmedicine.edu

Mirjana Maletic-Savatic, MD, PhD
Associate Professor, Departments of Pediatrics, Neurology, and Neuroscience
Baylor College of Medicine
Jan and Dan Duncan Neurological Research Institute at Texas Children Hospital
1250 Moursund St., Rm 1250, Houston, TX 77030
Tel 832-824-8807 Fax 832-825-8234 email: maletics@bcm.edu

Keywords: adult neurogenesis, neural stem cells, antibody, BASP1

Supplementary Figure 1.

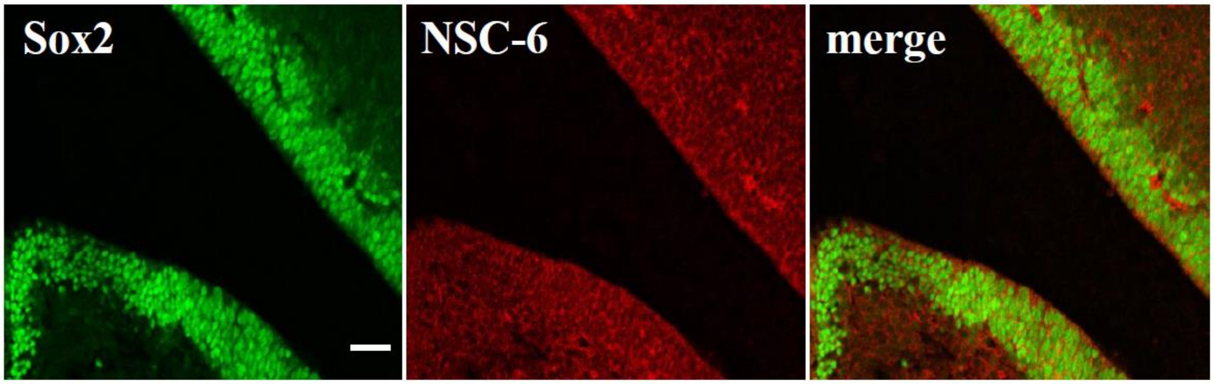

Supplementary Figure 2.

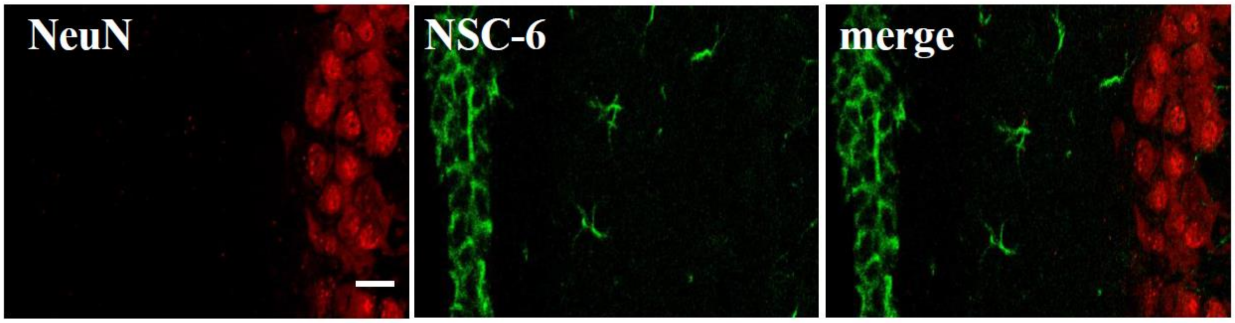

Supplementary Figure 3.

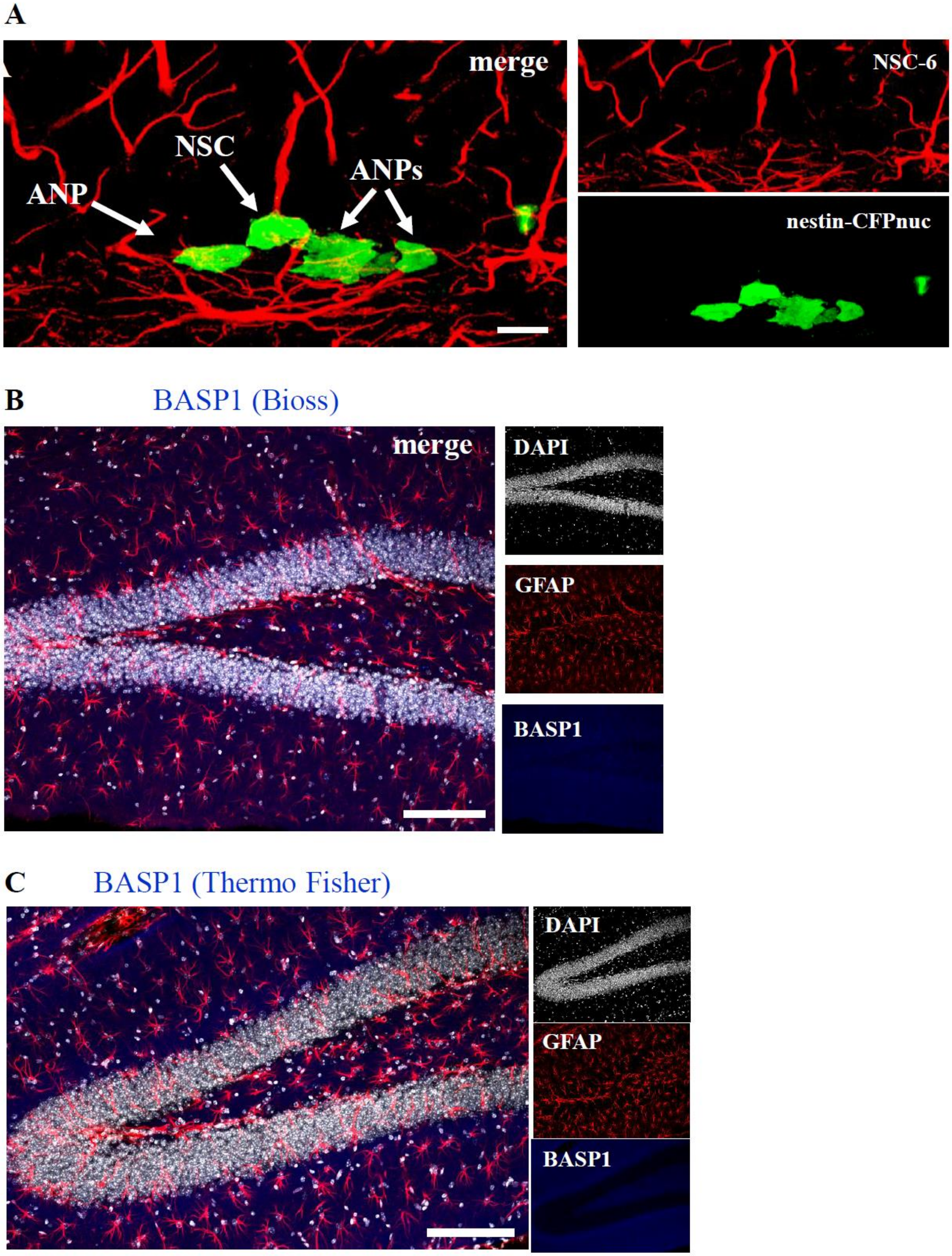

Supplementary Figure 4.

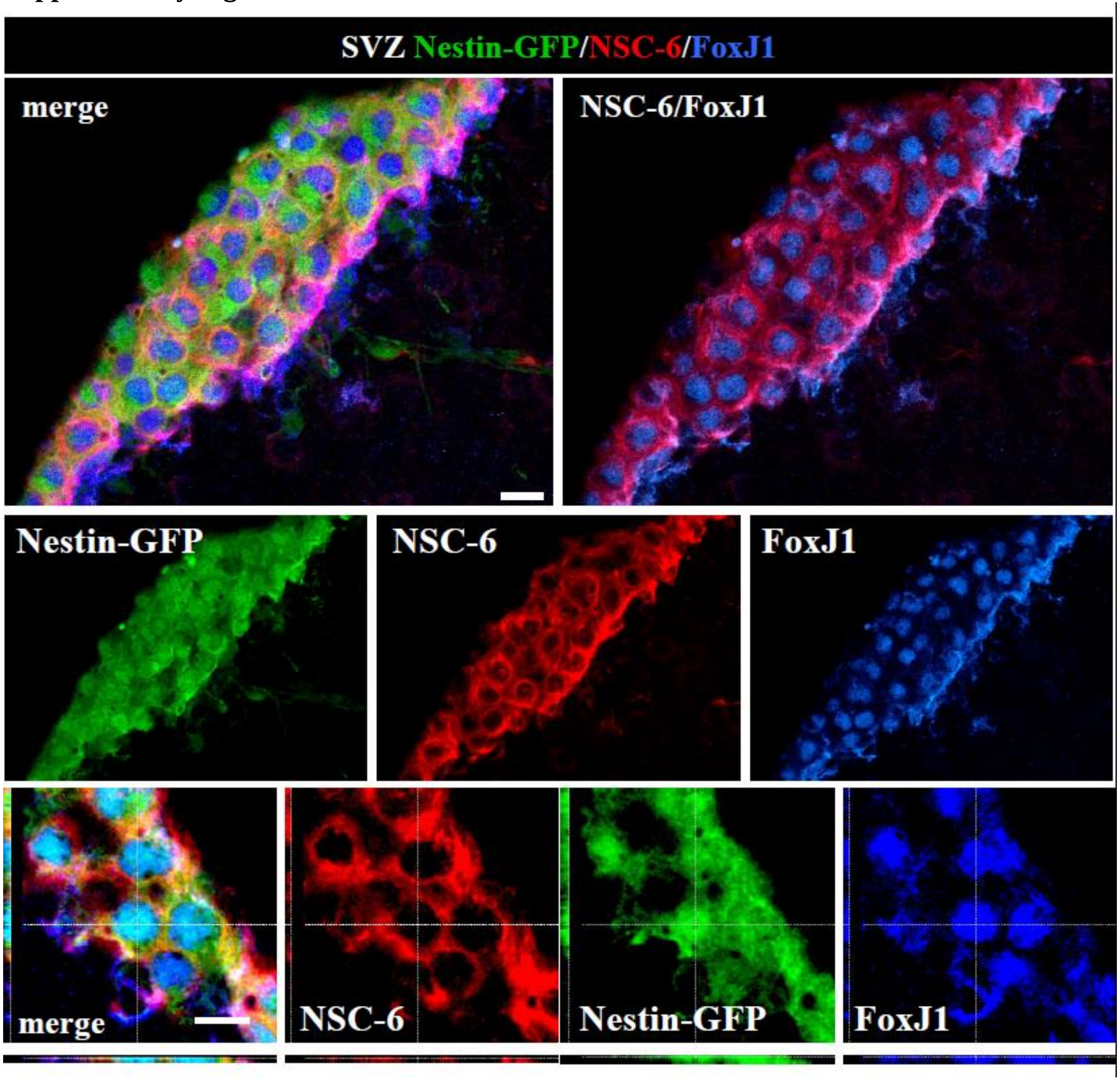

Supplementary Figure 5.

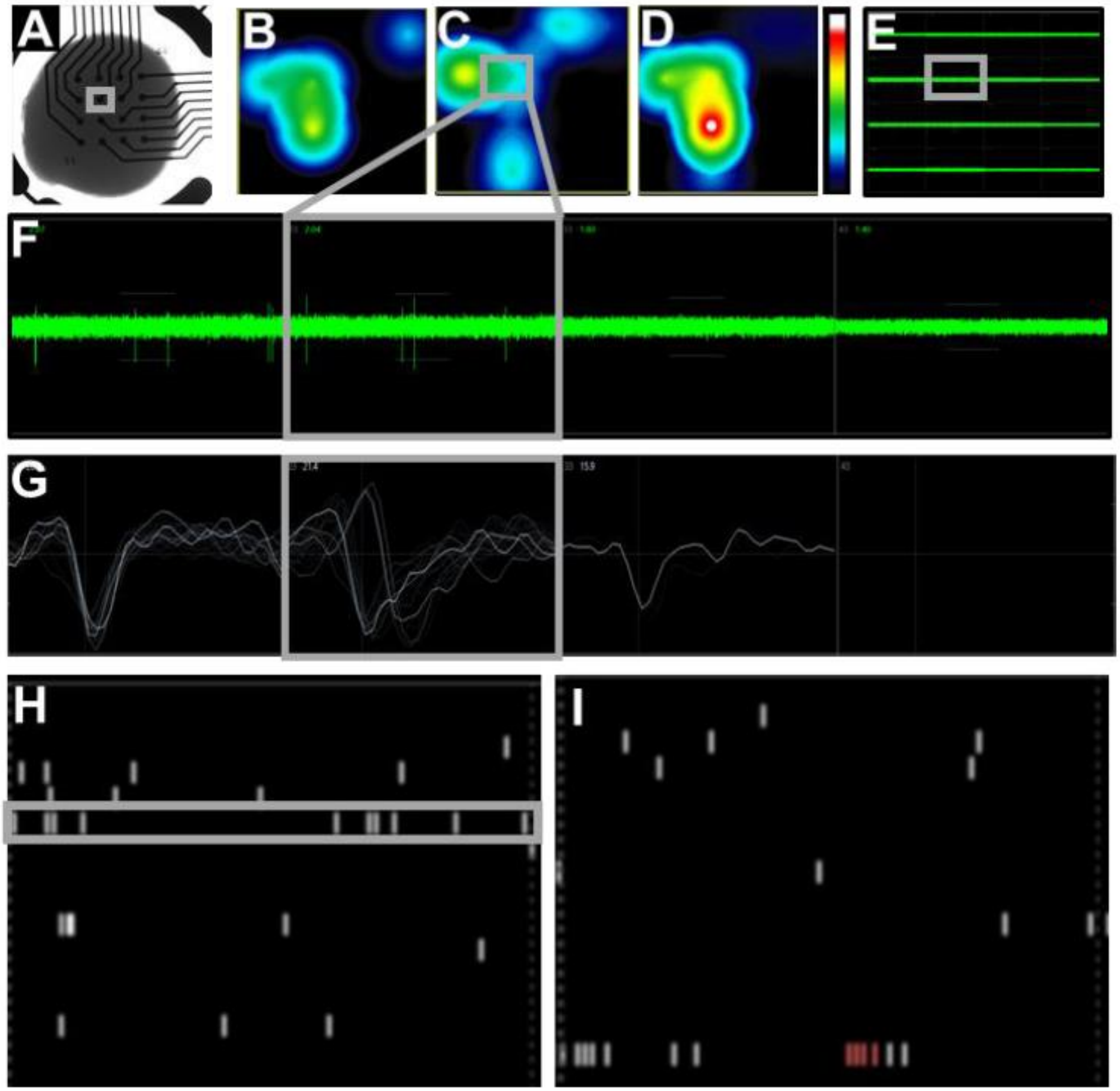

Supplementary Figure 1. NSC-6 antibody does not label Sox2+ amplifying neuroprogenitors in the subventricular zone of the embryonic mouse brain**.** Scale bar is 50 µm.

Supplementary Figure 2. NSC-6 antibody does not label mature NeuN+ neurons in the adult mouse parenchyma. Scale bar is 40 µm.

Supplementary Figure 3. (A) Immunolabeling with the NSC-6 antibody differentiates between NSCs and ANPs in the dentate gyrus of the *Nestin*-CFPnuc transgenic mice. Commercial BASP1 antibodies from Bioss **(B)** and Thermo Fisher **(C)** do not colocalize with GFAP and do not label NSCs in the dentate gyrus of a 3-month-old wild-type C57Bl6 mouse. Scale bar is 5 µm in A and 200 µm in B, C.

Supplementary Figure 4. NSC-6 immunolabeling in the SVZ colocalizes with FoxJ1, a marker of ependymal cells. *Nestin*-GFP staining **(A)**, NSC-6 staining **(B)**, FoxJ1 staining **(C)**, *Nestin*-GFP/NSC-6 /FoxJ1 **(D)** and NSC-6 /FoxJ1 **(E)**. Scale bar 10 µm.

Supplementary Figure 5. 4-month-old organoids exhibit spontaneous neuronal activities. **(A)** An organoid in a well of 48-well microelectrode array (MEA) plate. Gray square points to one electrode out of 16 in the well. (**B-D**) Heat maps show the neuronal activities detected by 16 electrodes at 3 timepoints (auto scaled, max 4 spikes/sec). **(E)** Neuronal activity detected by 16 electrodes over 500msec (max 20µV). **(F)** Continuous waveform plots show neuronal activity detected by 4 electrodes over 500 msec (max 20µV). Each spike represents an action potential from neurons around an electrode (average amplitude= 20.1±1.36µV and frequency=2.99±0.03 spikes/sec (mean ± SE, n=1275). **(G)** Spike plots show action potentials detected by 4 electrodes over 500msec (max 10µV). **(H)** Spike plot shows the spikes detected by 16 electrodes over 5sec. **(I)** Spike plot from a different organoid. The group of red spikes is a burst firing. A→C, E→H Boxes in corresponding figures represent recordings from the same electrode.
